# Tris(Butadiene) Compounds versus Butadiene Oligomerization in Second-Row Transition Metal Chemistry: Effects of Increased Ligand Fields

**DOI:** 10.3390/molecules26082220

**Published:** 2021-04-12

**Authors:** Yi Zhao, Qun Chen, Mingyang He, Zhihui Zhang, Xuejun Feng, Yaoming Xie, Robert Bruce King, Henry F. Schaefer

**Affiliations:** 1 School of Petrochemical Engineering, Changzhou University, Changzhou 213164, China; zhaoyi.ripp@sinopec.com (Y.Z.); chenqunjpu@yahoo.com (Q.C.); hmy@cczu.edu.cn (M.H.); zhangzh@cczu.edu.cn (Z.Z.); 2 Research Institute of Petroleum Processing (RIPP), SINOPEC, Beijing 100083, China; 3 Department of Chemistry and Center for Computational Quantum Chemistry, University of Georgia, Athens, GA 30602, USA; ymxie1@yahoo.com (Y.X.); ccq@uga.edu (H.F.S.)

**Keywords:** butadiene complexes, transition metals, density functional theory

## Abstract

The geometries, energetics, and preferred spin states of the second-row transition metal tris(butadiene) complexes (C_4_H_6_)_3_M (M = Zr–Pd) and their isomers, including the experimentally known very stable molybdenum derivative (C_4_H_6_)_3_Mo, have been examined by density functional theory. Such low-energy structures are found to have low-spin singlet and doublet spin states in contrast to the corresponding derivatives of the first-row transition metals. The three butadiene ligands in the lowest-energy (C_4_H_6_)_3_M structures of the late second-row transition metals couple to form a C_12_H_18_ ligand that binds to the central metal atom as a hexahapto ligand for M = Pd but as an octahapto ligand for M = Rh and Ru. However, the lowest-energy (C_4_H_6_)_3_M structures of the early transition metals have three separate tetrahapto butadiene ligands for M = Zr, Nb, and Mo or two tetrahapto butadiene ligands and one dihapto butadiene ligand for M = Tc. The low energy of the experimentally known singlet (C_4_H_6_)_3_Mo structure contrasts with the very high energy of its experimentally unknown singlet chromium (C_4_H_6_)_3_Cr analog relative to quintet (C_12_H_18_)Cr isomers with an open-chain C_12_H_18_ ligand.

## 1. Introduction

**Figure 1:**
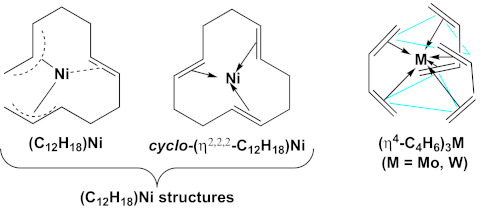
Comparison of the experimentally known nickel complexes with C_12_H_18_ ligands obtained from butadiene with the molybdenum and tungsten (C_4_H_6_)_3_M complexes with three separate butadiene ligands.

Butadiene transition metal chemistry dates back to the 1930 discovery of butadiene iron tricarbonyl by Reihlen et al. [1], its subsequent reinvestigation by Hallam and Pauson in 1958 [2] after the 1951 discovery of ferrocene [3,4], and its structural elucidation by Mills and Robinson [5] using X-ray crystallography. The next major development was the nickel-catalyzed trimerization of butadiene to give 1,5,9-cyclododecatriene. Proposed organonickel intermediates in this process include (η^3,2,3^-C_12_H_18_)Ni with an acyclic C_12_H_18_ ligand and cyclo-(η^2,2,2^-C_12_H_18_)Ni with a complexed 1,5,9-cyclododecatriene ligand (Figure 1). The latter intermediate has been structurally characterized by X-ray crystallography [6]. However, the instability of the former intermediate has precluded similar definitive structural characterization. The bonding of the central C=C double bond of the acyclic C_12_H_18_ ligand to the nickel atom in the former intermediate is uncertain since our recent theoretical studies [7] suggest a η^3,3^-hexahapto ligand rather than the originally proposed η^3,2,3^-octahapto ligand. No stable homoleptic (C_4_H_6_)*_n_*Ni (*n* = 2, 3) intermediates with unsubstituted separate butadiene ligands were observed in this system, even though the nickel atom in a hypothetical (C_4_H_6_)_2_Ni with two tetrahapto butadiene ligands would have the favored 18-electron configuration.

Synthesis of stable homoleptic butadiene metal complexes is found to require either substituted butadienes for the first-row transition metal derivatives or the second- and third-row transition metals for stable (C_4_H_6_)_3_M complexes of unsubstituted butadiene. It has been shown that 2,3-dimethylbutadiene forms an isolable crystalline homoleptic (2,3-MeC_4_H_4_)_2_Ni complex which is stable only below −10 °C [8]. Use of butadiene ligands with bulkier substituents leads to stable bis(butadiene)metal derivatives of first-row transition metals other than nickel. Thus, co-condensation of metal vapors with 1,4-bis(tert-butyl)butadiene gives corresponding [1,4-(Me_3_C)_2_C_4_H_4_]_2_M complexes (M = Ti [9], V [9], Co [10]). Similarly, the zerovalent 1,4-bis(trimethylsilyl)butadiene cobalt open sandwich [1,4-(Me_3_Si)_2_C_4_H_4_]_2_Co has been synthesized and structurally characterized [11].

In contrast to the first-row transition metals, the homoleptic tris(butadiene) complexes of the second- and third-row group 6 metals molybdenum and tungsten, (η^4^-C_4_H_6_)_3_M (M = Mo, W) are known as stable compounds. They can be synthesized by co-condensation of the metal vapors with butadiene [12] or by reduction of metal halides with activated magnesium in the presence of butadiene [13]. X-ray crystallography indicates trigonal prismatic coordination of the six C=C double bonds of the three butadiene ligands to the central metal atom [14]. Tris(butadiene)molybdenum and related substituted butadiene derivatives have been useful precursors to the synthesis of other zerovalent molybdenum compounds such as ditertiary phosphine derivatives of the type (diene)_2_Mo(diphosphane) [15] and the unusual Mo(GaC_5_Me_5_)_6_ cluster having a central molybdenum atom surrounded by six gallium atoms with octahedral coordination [16].

Although the tris(butadiene) derivatives of the heavier group 6 transition metals molybdenum and tungsten are stable compounds, the corresponding chromium derivative (C_4_H_6_)_3_Cr has never been synthesized. Furthermore, our recent theoretical studies [7] indicate that a singlet tris(butadiene)chromium structure, (η^4^-C_4_H_6_)_3_Cr, analogous to the stable molybdenum and tungsten derivatives, is energetically disfavored relative to a quintet (η^3,3^-C_12_H_18_)Cr structure with an open-chain bis(trihapto) C_12_H_18_ ligand. This observation suggests that the tris(butadiene) chemistry of the first-row transition metals is significantly different from that of their second- and third-row analogs. One factor may be the larger ligand field splitting in the second- and third-row transition metal complexes relative to their first-row transition metal analogs. An indication of this is the quintet ground state of the (C_12_H_18_)Cr system contrasted with the singlet state of the tris(butadiene) molybdenum and tungsten complexes.

In order to explore this matter further, we undertook density functional theory studies of the second-row transition metal butadiene complexes (C_4_H_6_)_3_M (M = Zr–Pd). In general, our results confirm low-spin structures to be the lowest-energy structures for all of the second-row transition metals in accord with the larger ligand field splitting in the second-row transition metal complexes relative to the corresponding first-row transition metal complexes. More specifically, our results confirm the experimental singlet (C_4_H_6_)_3_Mo structure to be the lowest-energy isomer by a significant margin in contrast to our earlier studies of the (C_4_H_6_)_3_Cr system. In addition, the low-energy low-spin (C_4_H_6_)_3_M structures of the second-row transition metals are found to exhibit an interesting trend. Thus, in (C_4_H_6_)_3_M complexes of the late transition metals requiring fewer electrons to attain the favored 18-electron configuration, the three butadiene ligands are found to couple to form a single open-chain C_12_H_18_ unit functioning as a hexahapto or octahapto ligand. However, for the early transition metals requiring more electrons to attain the favored 18-electron configuration, the three butadiene ligands remain as separate units thereby providing a total of 12 electrons for the central metal atom.

## 2. Theoretical Methods

Density functional theory (DFT) has been found to be a practical and effective computational tool, especially for organometallic compounds [17,18,19,20,21,22,23]. We used four DFT methods in this study. The first DFT method was the M06-L method, which has been claimed to be suitable for the study of organometallic and inorganic thermochemistry [24]. The second DFT method was the BP86 method, which combines Becke’s 1988 exchange functional with Perdew’s 1986 gradient corrected correlation functional [25,26]. Furthermore, in order to compare our current results with our earlier results on the first-row transition metal (C_4_H_6_)_3_M complexes [7], we also adopted the B3LYP [27,28] and B3LYP* [29] methods in the present paper. The results show that the four DFT methods predict similar results. The B3LYP and B3LYP* results are listed in the Supplementary Materials, and the M06-L results are those mainly discussed in the text. 

The Stuttgart double-ζ basis sets with an effective core potential (ECP) [30,31] were used for the second-row transition metals from zirconium to palladium. In these basis sets, 28 core electrons in the transition metal atoms are replaced by an effective core potential. This effective core approximation includes scalar relativistic contributions, which may become significant for heavy transition metal atoms. The valence basis sets are contracted from (8s7p6d) primitive sets to (6s5p3d). For the carbon and hydrogen atoms, the all-electron double-ζ plus polarization (DZP) basis sets were used. These are derived from Huzinaga and Dunning’s contracted double-ζ contraction set [32] by adding spherical harmonic polarization functions with the orbital exponents α_d_(C) = 0.75 and α_p_(H) = 0.75. All of the computations were performed using the Gaussian 09 program [33], in which the fine grid (75,302) is the default for evaluating integrals numerically [34].

The present paper discusses systems of the type (C_4_H_6_)_3_M, where M is a second-row transition metal. Thus, the (C_4_H_6_)_3_M (M = Zr, Mo, Ru, Pd) structures were optimized in singlet and triplet electronic states and the (C_4_H_6_)_2_M (M = Nb, Tc, Rh)—in doublet and quartet electronic states. The harmonic vibrational frequencies and the corresponding infrared intensities were determined at the same levels by evaluating force constants analytically.

The energetically low-lying (C_4_H_6_)_3_M species are shown in the figures. Each structure is designated as M-nZ, where M is the symbol of the central metal atom, n orders the structure according to their relative energies predicted by the M06-L method, and Z designates the spin states using S, D, T, and Q for the singlet, doublet, triplet, and quartet states, respectively.

## 3. Results and Discussion

### 3.1. (C_4_H_6_)_3_Pd

**Figure 2:**
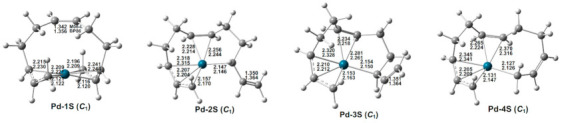
The optimized (C_4_H_6_)_3_Pd structures. The upper and lower distances (in Å) are from the M06-L and BP86 methods, respectively.

The four lowest-energy (C_4_H_6_)_3_Pd structures are all singlets with hexahapto straight-chain C_12_H_18_ ligands of various types, leaving one uncomplexed C=C double bond (Figure 2 and Table 1). This corresponds to a 16-electron configuration for the central palladium atom. In the lowest-energy (C_4_H_6_)_3_Pd structure Pd-1S, the three carbon atoms at each end of the C_12_ chain are bonded to the central palladium atom as trihapto allylic units leaving an uncomplexed C=C double bond of length 1.342 Å (M06-L) in the center of the chain. Structures Pd-2S and Pd-3S, lying 9.0 and 9.1 kcal/mol (M06-L), respectively, in energy above Pd-1S, have similar geometries with a hexahapto η^3,2,1^-C_12_H_18_ ligand. In Pd-2S and Pd-3S, a terminal allylic unit, the central C=C double bond, and an interior carbon atom from the other terminal allylic unit are all within bonding distance of the palladium atom. The difference between Pd-2S and Pd-3S is the orientation of their terminal uncomplexed C=C double bonds. Structure Pd-4S, with a hexahapto η^3,2,1^-C_12_H_18_ ligand, lies 9.5 kcal/mol in energy above Pd-1S. The hexahapto coordination includes a terminal allylic unit at one end of the C_12_ chain and the carbon atom at the other end of the C_12_ chain in addition to the central C=C double bond. These four structures correspond to the four lowest-energy structures for the nickel (C_4_H_6_)_3_Ni system [7] with similar relative energies (Table 1).

### 3.2. (C_4_H_6_)_3_Rh

**Figure 3:**
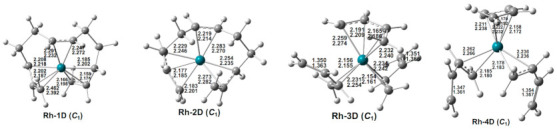
Optimized (C_4_H_6_)_3_Rh structures. The upper and lower distances (in Å) are from the M06-L and BP86 methods, respectively.

Four low-energy (C_4_H_6_)_3_Rh doublet structures were found within ~17 kcal/mol of the lowest-energy structure Rh-1D (Figure 3 and Table 2). In the two lowest-energy (C_4_H_6_)_3_Rh structures Rh-1D and Rh-2D, the three butadiene ligands are coupled to form a C_12_H_18_ chain. Structure Rh-1D has an octahapto η^3,2,3^-C_12_H_18_ ligand, thereby giving the central rhodium atom a 17-electron configuration. The second (C_4_H_6_)_3_Rh structure Rh-2D, lying 8.9 kcal/mol (M06-L) in energy above Rh‑1D, has a hexahapto η^3,2,1^-C_12_H_18_ ligand with an uncomplexed C=C double bond at the end of the C_12_H_18_ chain.

The significantly higher-energy (C_4_H_6_)_3_Rh structures Rh-3D and Rh-4D, lying 14.6 and 16.5 kcal/mol (M06-L), respectively, in energy above Rh-1D, have three separate butadiene ligands. One of these butadiene ligands is a tetrahapto ligand, whereas the two remaining butadiene ligands are dihapto ligands. Structures Rh-3D and Rh-4D differ only in the relative orientations of their terminal uncomplexed C=C double bonds.

The previous DFT study [7] of the analogous cobalt system (C_4_H_6_)_3_Co found quartet structures with the lowest-energy quartet structure lying ~15 kcal/mol above the lowest-energy isomer, which is a doublet. The lowest-energy quartet structure for (C_4_H_6_)_3_Rh structure is a very high-energy structure, lying more than 29 kcal/mol in energy above Rh-1D. Thus, quartet (C_4_H_6_)_3_Rh structures do not appear to be chemically relevant and therefore are not discussed in detail in this paper. The high energy of the quartet (C_4_H_6_)_3_Rh structures as compared with the analogous cobalt system is a consequence of the higher ligand field strengths in the second-row transition metal complexes as compared with the analogous first-row transition metal complexes.

### 3.3. (C_4_H_6_)_3_Ru

**Figure 4:**
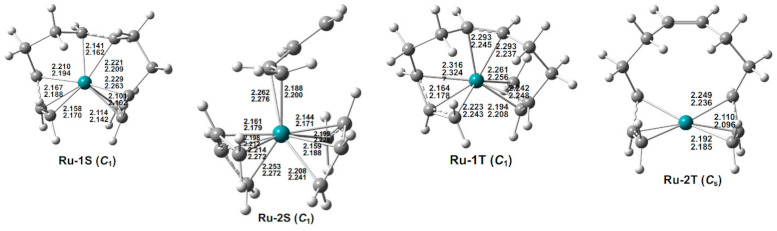
The optimized (C_4_H_6_)_3_Ru structures. The upper and lower distances (in Å) are from the M06-L and BP86 methods, respectively.

Four low-energy (C_4_H_6_)_3_Ru structures were optimized, namely, two singlets and two triplets (Figure 4 and Table 3). The lowest-energy (C_4_H_6_)_3_Ru structure Ru-1S has a singlet spin state with an octahapto η^3,2,3^-C_12_H_18_ ligand, thereby providing the ruthenium atom with a 16-electron configuration. A second singlet (C_4_H_6_)_3_Ru structure Ru-2S, lying only 4.3 kcal/mol (M06-L) in energy above Ru-1S, has three separate butadiene ligands. Two of the butadiene ligands in Ru-2S are tetrahapto ligands, whereas the third butadiene ligand is only a dihapto ligand. This gives the central ruthenium atom the favored 18-electron configuration.

The lowest-energy triplet (C_4_H_6_)_3_Ru structures lie ~20 kcal/mol in energy above the lowest-energy singlet structure Ru-1S (Figure 4 and Table 3). This contrasts with the analogous (C_4_H_6_)_3_Fe system for which the lowest-energy structures are triplet spin state structures [7]. Furthermore, for (C_4_H_6_)_3_Fe, even a quintet structure has a lower energy than the lowest-energy singlet structure. This, again, is an example of the preference of the second-row transition metals for lower spin states relative to analogous complexes of the first-row transition metals. The triplet (C_4_H_6_)_3_Ru structure Ru-1T has an octahapto η^3,2,3^-C_12_H_18_ ligand, thereby giving the ruthenium atom a 16-electron configuration consistent with a triplet spin state in a high-spin complex. The second triplet (C_4_H_6_)_3_Ru structure Ru-2T with a similar energy as Ru-1T has a hexahapto η^3,3^-C_12_H_18_ ligand similar to that in Pd-1S, with an uncomplexed central C=C double bond. This gives the ruthenium atom in Ru-2T only a 14-electron configuration, which can also be the basis for a triplet spin state structure. Note that the M06-L method predicts Ru-1T and Ru-2T to have essentially the same energies, while the BP86 method predicts Ru-2T to lie ~7 kcal/mol in energy above Ru-1T.

### 3.4. (C_4_H_6_)_3_Tc

**Figure 5:**
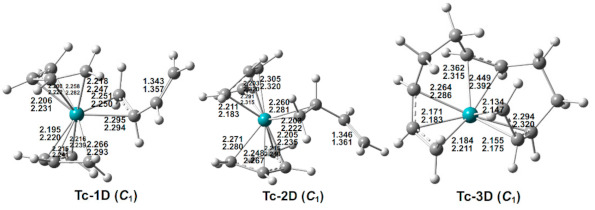
The optimized (C_4_H_6_)_3_Tc structures. The upper and lower distances (in Å) are from the M06-L and BP86 methods, respectively.

Three low-energy doublet structures were optimized for (C_4_H_6_)_3_Tc (Figure 5 and Table 4). Quartet and sextet (C_4_H_6_)_3_Tc structures have energies at least 13 kcal/mol in energy above the lowest-energy doublet (C_4_H_6_)_3_Tc structure Tc-1D. This contrasts with the (C_4_H_6_)_3_Mn system for which the lowest energy structures are sextet structures and the lowest- energy doublet structure lies ~24 kcal/mol in energy above the lowest-energy sextet structure [7]. This, again, is an indication of the higher ligand field strength in the second-row transition metals relative to analogous complexes of the first-row transition metals.

The lowest-energy (C_4_H_6_)_3_Tc structures Tc-1D and Tc-2D are almost degenerate in energy within 1.0 kcal/mol (M06-L) (Figure 5 and Table 4). Both structures have two tetrahapto butadiene ligands and one dihapto butadiene ligand with an uncomplexed C=C double bond of length ~1.35 Å. This arrangement of butadiene ligands gives the technetium atoms in these structures 17-electron configurations, consistent with the doublet spin state. Structures Tc-1D and Tc-2D differ only in the orientations of their butadiene ligands. The other doublet (C_4_H_6_)_3_Tc structure Tc-3D, lying 11.3 kcal/mol (M06-L) in energy above Tc-1D, has three butadiene units coupled to form an octahapto η^3,2,3^-C_12_H_18_ ligand, thereby giving the technetium atom a 15-electron configuration.

### 3.5. (C_4_H_6_)_3_Mo

**Figure 6:**
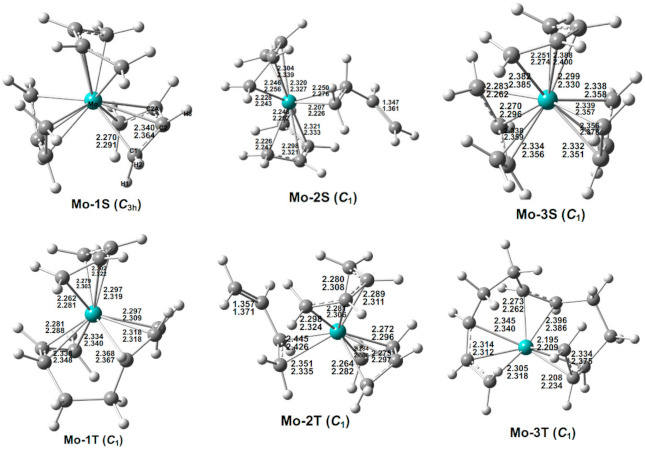
The optimized (C_4_H_6_)_3_Mo structures. The upper and lower distances (in Å) are from the M06-L and BP86 methods, respectively.

Six low-energy (C_4_H_6_)_3_Mo structures were found, namely, three singlets and three triplets (Figure 6 and Table 5). The theoretical singlet structure Mo-1S is predicted to be the global minimum, and is experimentally known as a stable species [12,13,14,15]. Structure Mo‑1S has three separate tetrahapto butadiene ligands, thereby giving the molybdenum atom the favored 18‑electron configuration. Table 6 shows that our predicted geometric parameters agree with experiment, especially with the more reliable low-temperature X‑ray crystallographic structures in the 2002 paper [14]. For example, the predicted Mo–C1 distances (~2.28 Å) and the Mo–C2 distances (~2.35 Å) agree within 0.02 Å. The C1–C2 distances (~1.44 Å) and C2–C2A distances (~1.41 Å) also agree within 0.02 Å. Our predicted C1-C2-C2A angle (118.9° or 119.1°) agrees with the experimental result (119.7°) within 1°. Kaupp et al. also carried out a computational study on Mo-1S [14]. We found that the geometry parameters from different computational methods are in reasonable agreement (Table S8 in the Supporting Information).

The singlet (C_4_H_6_)_3_Mo structure Mo-2S, with two tetrahapto butadiene ligands and one dihapto butadiene ligand similar to Ru-2S and Tc-1D, is predicted to lie 12.8 kcal/mol (M06-L) in energy above Mo-1S. The molybdenum atom in Mo-2S has a 16-electron configuration. The singlet Mo-3S, lying 13.3 kcal/mol (M06-L) in energy above Mo-1S, has a geometry similar to that of Mo-1S, i.e., with three tetrahapto butadiene ligands, but in a different orientation. Thus, in Mo-3S, the molybdenum atom has the favored 18-electron configuration.

In contrast to their molybdenum analogs, the singlet (C_4_H_6_)_3_Cr structures are high-energy structures relative to the triplet and especially the quintet state (C_4_H_6_)_3_Cr isomers [7]. This difference is another example of the increased ligand field strength of the second-row transition metal complexes relative to the corresponding first-row transition metal complexes, leading to lower-spin state preferred structures for the second-row transition metals. Furthermore, the high energy of singlet (η^4^-C_4_H_6_)_3_Cr relative to higher-spin state isomers with coupled butadiene ligands as compared with the low energy of its molybdenum analog (η^4^-C_4_H_6_)_3_Mo explains why the chromium derivative is unknown but the molybdenum structure is a stable species.

The lowest-energy triplet (C_4_H_6_)_3_Mo structure Mo-1T, lying 16.5 kcal/mol (M06-L) in energy above Mo-1S, has a coupled straight-chain η^3,3^-C_8_H_12_ ligand and a separate η^4^-C_4_H_6_ ligand, thereby giving the molybdenum atom a 16-electron configuration consistent with the triplet spin state (Figure 6 and Table 5). The second triplet (C_4_H_6_)_3_Mo structure Mo-2T, lying 29.3 kcal/mol (M06-L) in energy above Mo-1S, has a ligand arrangement similar to Mo-2S with two tetrahapto η^4^-C_4_H_6_ ligands and one dihapto η^2^-C_4_H_6_ ligand. This gives the molybdenum atom in Mo-2T a 16-electron configuration similar to Mo-2S. Thus, Mo-2T can be regarded as the high-spin analog of Mo-2S. The significantly higher-energy triplet structure Mo-3T, lying 36.2 kcal/mol (M06-L) in energy above Mo-1S, has a long-chain octahapto η^3,2,3^-C_12_H_18_ ligand, thereby giving the molybdenum atom a 14-electron configuration.

### 3.6. (C_4_H_6_)_3_Nb

**Figure 7:**
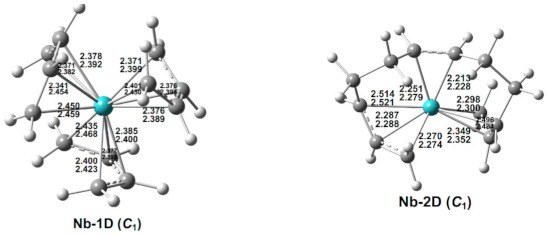
The optimized (C_4_H_6_)_3_Nb structures. The upper and lower distances (in Å) are from the M06-L and BP86 methods, respectively.

Two low-energy doublet (C_4_H_6_)_3_Nb structures were found (Figure 7 and Table 7). The lowest-energy (C_4_H_6_)_3_Nb structure Nb-1D has three separate tetrahapto butadiene ligands, thereby giving the niobium atom a 17-electron configuration consistent with the doublet spin state. The second doublet (C_4_H_6_)_3_Nb structure Nb-2D, lying 10.7 kcal/mol (M06-L) in energy above Nb-1D, has a coupled long-chain η^3,2,3^-C_12_H_18_ ligand, thereby giving the niobium atom a 13-electron configuration (Figure 7 and Table 7). Quartet (C_4_H_6_)_3_Nb structures are high-energy structures lying at least 26 kcal/mol in energy above Nb-1D and thus are not considered in detail. This contrasts with the (C_4_H_6_)_3_V system for which quartet structures are the lowest-energy structures.

### 3.7. (C_4_H_6_)_3_Zr

**Figure 8:**
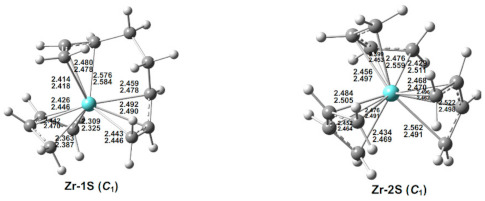
The optimized (C_4_H_6_)_3_Zr structures. The upper and lower distances (in Å) are from the M06-L and BP86 methods, respectively.

Two low-energy (C_4_H_6_)_3_Zr structures were found (Figure 8 and Table 8). The global minimum by M06-L is the singlet structure Zr-1S. In Zr-1S, the Zr–C distances clearly indicate that one of the C_4_H_6_ ligands is tetrahapto, and the remaining two C_4_H_6_ ligands are coupled to form an acyclic η^3,3^-C_8_H_12_ ligand. The other singlet structure Zr-2S, lying 8.9 kcal/mol (M06-L) in energy above Zr-1S, is shown by its Zr–C distances to have three separate tetrahapto η^4^-C_4_H_6_ ligands, thereby giving the zirconium atom a 16-electron configuration. The lowest energy triplet (C_4_H_6_)_3_Zr structure lies ~27 kcal/mol in energy above Zr-1S and therefore is not considered in detail.

## 4. Summary

The lowest-energy (C_4_H_6_)_3_M structures for the second-row transition metals from zirconium to palladium are all low-spin singlet and doublet structures, in contrast to the corresponding derivatives of the first-row transition metals. This is a reflection of the higher ligand field strength of the second-row transition metal derivatives relative to the analogous first-row transition metal derivatives.

In addition to the preference for low-spin structures, the energetically preferred structure types for the second-row transition metal (C_4_H_6_)_3_M derivatives are found to depend on the electronic requirements of the central metal atom to attain the favored 18-electron configuration. Thus, attaining the 18-electron configuration for the late second-row transition metals from palladium to ruthenium leaves one or two uncomplexed C=C double bonds in the set of three surrounding butadiene ligands. In such complexes, the uncomplexed C=C double bonds provide reactive sites to couple with adjacent butadiene ligands to form structures with an open-chain C_12_H_18_ ligand. As a result, the three butadiene ligands in the lowest-energy (C_4_H_6_)_3_M structures of the late second-row transition metals couple to form a C_12_H_18_ ligand that binds to the central metal atom as a hexahapto ligand for M = Pd but as an octahapto ligand for M = Rh and Ru. Thus, the four lowest-energy (C_12_H_18_)Pd structures resemble their nickel analogs with similar relative energies. However, attaining or even approaching the favored 18-electron configuration for (C_4_H_6_)_3_M complexes of the early transition metals from zirconium to technetium requires complexation of all six C=C double bonds of three surrounding butadiene ligands. As a result, the lowest-energy (C_4_H_6_)_3_M structures have three tetrahapto butadiene ligands for M = Zr, Nb, and Mo or two tetrahapto butadiene ligands and one dihapto butadiene ligand for M = Tc. The low energy of the experimentally known singlet (C_4_H_6_)_3_Mo structure contrasts with the very high energy of its experimentally unknown singlet chromium (C_4_H_6_)_3_Cr analog relative to quintet (C_12_H_18_)Cr isomers with an open-chain C_12_H_18_ ligand. The (C_4_H_6_)_3_M complexes studied in this work are potentially accessible by reactions of the metal vapors with butadiene under suitable conditions similar to the reported synthesis of the molybdenum derivative (C_4_H_6_)_3_Mo.

## Figures and Tables

**Table 1:** Total energies with ZPVE (zero potential vibrational energy) correction (E in hartrees), relative energies with ZPVE corrections (ΔE in kcal/mol), relative enthalpies (ΔH in kcal/mol), and relative free energies (ΔG_298_ in kcal/mol) for the (C_4_H_6_)_3_Pd structures; comparison with the (C_4_H_6_)_3_Ni system [7].

		Pd-1S (C_1_)	Pd-2S (C_1_)	Pd-3S (C_1_)	Pd-4S (C_1_)
M06-L	E	−595.76525	−595.75132	−595.75118	−595.75072
	∆E (∆H)	0.0 (0.0)	9.0 (9.0)	9.1 (8.9)	9.5 (9.3)
	∆G_298_	0.0	8.2	8.9	9.4
BP86	E	−595.85507	−595.84924	−595.84701	−595.84689
	∆E (∆H)	0.0 (0.0)	3.6 (3.7)	5.0 (5.0)	5.0 (5.1)
	∆G_298_	0.0	2.8	4.5	4.7
(C_4_H_6_)_3_Ni (B3LYP*)	∆E	0.0	6.7	8.9	9.5

**Table 2:** Total energies with ZPVE correction (E in hartrees), relative energies with ZPVE corrections (ΔE in kcal/mol), relative enthalpies (ΔH in kcal/mol), and relative free energies (ΔG_298_ in kcal/mol) for the (C_4_H_6_)_3_Rh structures.

		Rh-1D (C_1_)	Rh-2D (C_1_)	Rh-3D (C_1_)	Rh-4D (C_1_)
M06-L	E	−578.40533	−578.39125	−578.38207	−578.37905
	∆E (∆H)	0.0 (0.0)	8.9 (8.9)	14.6 (16.4)	16.5 (18.1)
	∆G_298_	0.0	9.0	11.8	14.2
BP86	E	−578.52088	−578.50221	−578.49830	−578.49439
	∆E (∆H)	0.0 (0.0)	11.6 (11.8)	14.1 (15.8)	16.5 (18.2)
	∆G_298_	0.0	11.6	11.5	14.1

**Table 3:** Total energies with ZPVE corrections (E in hartrees), relative energies with ZPVE corrections (ΔE in kcal/mol), relative enthalpies (ΔH in kcal/mol), and relative free energies (ΔG_298_ in kcal/mol) for the (C_4_H_6_)_3_Ru structures; comparison with the (C_4_H_6_)_3_Fe system [7].

		Ru-1S (C_1_)	Ru-2S (C_1_)	Ru-1T (C_1_)	Ru-2T (C_s_)
M06-L	E	−562.75206	−562.74555	−562.71903	−562.72023
	∆E (∆H)	0.0 (0.0)	4.3 (5.1)	20.7 (21.3)	20.2 (20.7)
	∆G_298_	0.0	3.4	19.0	18.0
BP86	E	−562.87730	−562.86819	−562.84327	−562.83165
	∆E (∆H)	0.0 (0.0)	6.0 (6.6)	21.4 (21.7)	28.7 (29.2)
	∆G_298_	0.0	5.4	20.7	26.8
(C_4_H_6_)_3_Fe (B3LYP*)	∆E	9.8	31.0	0.0	0.9

**Table 4:** Total energies with ZPVE corrections (E in hartrees), relative energies with ZPVE corrections (ΔE in kcal/mol), relative enthalpies (ΔH in kcal/mol), and relative free energies (ΔG_298_ in kcal/mol) for the (C_4_H_6_)_3_Tc structures; comparison with the (C_4_H_6_)_3_Mn system [7].

		Tc-1D (C_1_)	Tc-2D (C_1_)	Tc-3D (C_1_)
M06-L	E	−548.63293	−548.63130	−548.61490
	∆E (∆H)	0.0 (0.0)	1.0 (0.8)	11.3 (10.4)
	∆G_298_	0.0	1.3	12.8
BP86	E	−548.76092	−548.76018	−548.73899
	∆E (∆H)	0.0 (0.0)	0.5 (0.3)	13.8 (12.4)
	∆G_298_	0.0	0.6	15.5
(C_4_H_6_)_3_Mn (B3LYP*)	∆E	23.9	25.9	27.5

**Table 5:** Total energies with ZPVE corrections (E in hartrees), relative energies with ZPVE corrections (ΔE in kcal/mol), relative enthalpies (ΔH in kcal/mol), and relative free energies (ΔG_298_ in kcal/mol) for the (C_4_H_6_)_3_Mo structures; comparison with the (C_4_H_6_)_3_Cr system [7].

		Mo-1S (C_3h_)	Mo-2S (C_1_)	Mo-3S (C_1_)	Mo-1T (C_1_)	Mo-2T (C_1_)	Mo-3T (C_s_)
M06-L	E	−536.04474	−536.02426	−536.02360	−536.01838	−535.99805	−535.98708
	∆E (∆H)	0.0 (0.0)	12.8 (13.5)	13.3 (13.5)	16.5 (17.0)	29.3 (30.5)	36.2 (36.8)
	∆G_298_	0.0	10.9	12.2	14.2	25.9	34.3
BP86	E	−536.16875	−536.15267	−536.14640	−536.14416	−536.12279	−536.11094
	∆E (∆H)	0.0 (0.0)	10.1 (10.7)	14.0 (14.3)	15.4 (15.8)	28.8 (30.0)	36.3 (36.2)
	∆G_298_	0.0	8.1	12.9	13.1	25.4	34.3
(C_4_H_6_)_3_Cr (B3LYP*)	∆E	−	28.5	39.5	2.3	15.3	17.4

**Table 6:** Computed structural parameters of Mo-1S compared to experimental data ^a^.

	M06-L ^b^	BP86 ^b^	Expt ^c^	Expt ^d^	Expt ^e^
r(Mo-C1)	2.270	2.291	2.29 (1)	2.301 (8)	2.284 (2), 2.273 (6)
r(Mo-C2)	2.340	2.364	2.29 (1)	2.317 (7)	2.325 (2), 2.330 (5)
r(C1-C2)	1.430	1.443	1.32 (2)	1.336 (11)	1.414 (4), 1.414 (7)
r(C2-C2A)	1.402	1.414	1.55 (3)	1.560 (18)	1.403 (5), 1.388 (9)
r(C1-H1)	1.092	1.100	1.14 (8)		
r(C1-H2)	1.088	1.097	0.96 (10)		
r(C2-H3)	1.088	1.098	1.12 (10)		
∠C1-C2-C2A	118.9	119.1	114.84 (88), 119.20 (15)		119.7 (4)
∠H1-C1-C2	117.2	117.6	121.99 (5.30)		
∠H2-C1-C2	117.5	117.1	119.10 (6.33)		
∠H3-C2-C2A	119.0	118.9	125.24 (5.32)		
Σ∠(C1) ^f^	348.6	348.6	349.9		

^a^ Distances in Å, angles in degrees. ^b^ The present work. ^c^ Skell, P.S.; McGlinchey, M.J. *Angew. Chem. Int. Ed.* **1975**, *14*, 195. ^d^ Green, J.C.; Kelly, M.R.; Grebenik, P.D.; Briant, C.E.; McEvoy, N.A.; Mingos, D.M.P. *J. Organomet. Chem.* **1982**, *228*, 239. ^e^ Ref. [14]. ^f^ Sum of angles around C1 (the terminal carbon).

**Table 7:** Total energies with ZPVE corrections (E in hartrees), relative energies with ZPVE corrections (ΔE in kcal/mol), relative enthalpies (ΔH in kcal/mol), and relative free energies (ΔG_298_ in kcal/mol) for the (C_4_H_6_)_3_Nb structures; comparison with the (C_4_H_6_)_3_V system [7].

		Nb-1D (C_1_)	Nb-2D (C_1_)
M06-L	E	−524.79243	−524.77533
	∆E (∆H)	0.0 (0.0)	10.7 (9.8)
	∆G_298_	0.0	11.5
BP86	E	−524.91119	−524.89914
	∆E (∆H)	0.0 (0.0)	7.6 (6.7)
	∆G_298_	0.0	8.1
(C_4_H_6_)_3_V (B3LYP)	∆E	9.8	4.4

**Table 8:** Total energies with ZPVE correction (E in hartrees), relative energies with ZPVE correction (ΔE in kcal/mol), relative enthalpies (ΔH in kcal/mol), and relative free energies (ΔG_298_ in kcal/mol) for the (C_4_H_6_)_3_Zr structures.

		Zr-1S (C_1_)	Zr-2S (C_1_)
M06-L	E	−514.89112	−514.87692
	∆E (∆H)	0.0 (0.0)	8.9 (9.4)
	∆G_298_	0.0	8.5
BP86	E	−515.00107	−514.98811
	∆E (∆H)	0.0 (0.0)	8.1 (8.5)
	∆G_298_	0.0	8.1
(C_4_H_6_)_3_Ti (B3LYP)	∆E	0.0	15.5

## Data Availability

Not applicable.

## Supplementary Materials

The following are available online, Tables S1–S25: Atomic coordinates of the optimized structures for the (C_4_H_6_)_3_M (M = Pd, Rh, Ru, Tc, Mo, Nb, Zr) complexes.
