# Supplementary material for: Tris(Butadiene) Compounds versus Butadiene Oligomerization in Second-Row Transition Metal Chemistry: Effects of Increased Ligand Fields
Source: Molecules. 2021 Apr 12;26(8):2220. doi: 10.3390/molecules26082220 (PMC8068848; doi:10.3390/molecules26082220)
Supplement: Supplementary file 1 [file molecules-26-02220-s001.pdf]

Tris(butadiene) Compounds Versus Butadiene Oligomerization in Second Row Transition Metal Chemistry: Effects of Increased Ligand Fields

Yi Zhao,[b] Qun Chen,[a] Mingyang He,[a] Zhihui Zhang,[a] Xuejun Feng,[a,c]*
Yaoming Xie,[c] R. Bruce King,[c]* and Henry F. Schaefer[c]

[a] *School of Petrochemical Engineering, Changzhou University, Changzhou, People's Republic of China 213164*

[b]*SINOPEC Research Institute of Petroleum Processing (RIPP), Beijing, People's Republic of China 100083*

[c]*Department of Chemistry and Center for Computational Quantum Chemistry University of Georgia, Athens, Georgia 30602, USA*

Supporting Information

Tables S1-S7. Total energies with ZPVE correction (E in hartree), relative energies with ZPVE corrections (ΔE in kcal/mol), relative thermal Energies(ΔH in kcal/mol), and relative free energies (ΔG298 in kcal/mol) for the $(C_4H_6)_3M$ (M = Pd, Rh, Ru, Tc, Mo, Nb,Zr) structures.

Table S8. Our theoretical structural parameters of $(C_4H_6)_3Mo$ compared with those from Kaupp *et al.*[14]

Tables S9-S33. The optimized geometries for the $(C_4H_6)_3M$ (M = Pd, Rh, Ru, Tc, Mo, Nb, Zr) structures.

Table S1. Total energies with ZPVE correction (E in hartree), relative energies with ZPVE corrections (ΔE in kcal/mol), relative thermal Energies(ΔH in kcal/mol), and relative free energies (ΔG_{298} in kcal/mol) for the $(C_4H_6)_3Pd$ structures; comparison with the $(C_4H_6)_3Ni$ system.

		**Pd-1S** (C_1)	**Pd-2S** (C_1)	**Pd-3S** (C_1)	**Pd-4S** (C_1)
M06-L	E	-595.76525	-595.75132	-595.75118	-595.75072
	ΔE(ΔH)	0.0(0.0)	9.0(9.0)	9.1(8.9)	9.5(9.3)
	ΔG_{298}	0.0	8.2	8.9	9.4
BP86	E	-595.85507	-595.84924	-595.84701	-595.84689
	ΔE(ΔH)	0.0(0.0)	3.6(3.7)	5.0(5.0)	5.0(5.1)
	ΔG_{298}	0.0	2.8	4.5	4.7
B3LYP*	E	-595.41694	-595.40933	-595.40752	-595.40738
	ΔE(ΔH)	0.0(0.0)	4.8(4.9)	5.9(5.9)	6.0(5.7)
	ΔG_{298}	0.0	4.2	5.6	5.7
B3LYP	E	-595.77595	-595.76813	-595.76634	-595.76622
	ΔE(ΔH)	0.0(0.0)	4.4(5.0)	5.8(6.0)	5.6(6.2)
	ΔG_{298}	0.0	4.3	5.8	5.8
$(C_4H_6)_3Ni$ (B3LYP*)	ΔE	0.0	6.7	8.9	9.5

Table S2. Total energies with ZPVE correction (E in hartree), relative energies with ZPVE corrections (ΔE in kcal/mol), relative thermal Energies(ΔH in kcal/mol), and relative free energies (ΔG_{298} in kcal/mol) for the $(C_4H_6)_3Rh$ structures.

		**Rh-1D** (C_1)	**Rh-2D**(C_1)	**Rh-3D** (C_1)	**Rh-4D** (C_1)
M06-L	E	-578.40533	-578.39125	-578.38207	-578.37905
	ΔE(ΔH)	0.0(0.0)	8.9(8.9)	14.6(16.4)	16.5(18.1)
	ΔG_{298}	0.0	9.0	11.8	14.2
BP86	E	-578.52088	-578.50221	-578.49830	-578.49439
	ΔE(ΔH)	0.0(0.0)	11.6(11.8)	14.1(15.8)	16.5(18.2)
	ΔG_{298}	0.0	11.6	11.5	14.1
B3LYP*	E	-578.07248	-578.05603	-578.05298	-578.04891
	ΔE(ΔH)	0.0(0.0)	10.3(10.4)	12.2(14.0)	14.8(16.4)
	ΔG_{298}	0.0	10.2	9.5	12.2
B3LYP	E	-578.42540	-578.40956	-578.40560	-578.40159
	ΔE(ΔH)	0.0(0.0)	9.9(10.1)	12.4(14.2)	14.9(16.6)
	ΔG_{298}	0.0	9.5	9.7	12.3

Table S3. Total energies with ZPVE corrections (E in hartree), relative energies with ZPVE corrections (ΔE in kcal/mol), relative thermal Energies(ΔH in kcal/mol), and relative free energies (ΔG_{298} in kcal/mol) for the $(C_4H_6)_3Ru$ structures; comparison with the $(C_4H_6)_3Fe$ system.Error! Bookmark not defined..

		**Ru-1S** (C_1)	**Ru-2S** (C_1)	**Ru-1T** (C_1)	**Ru-2T** (C_s)
M06-L	E	-562.75206	-562.74555	-562.71903	-562.72023
	ΔE(ΔH)	0.0(0.0)	4.3(5.1)	20.7(21.3)	20.2(20.7)
	ΔG_{298}	0.0	3.4	19.0	18.0
BP86	E	-562.87730	-562.86819	-562.84327	-562.83165
	ΔE(ΔH)	0.0(0.0)	6.0(6.6)	21.4(21.7)	28.7(29.2)
	ΔG_{298}	0.0	5.4	20.7	26.8
B3LYP*	E	-562.42605	-562.41641	-562.39660	-562.39283
	ΔE(ΔH)	0.0(0.0)	6.0(7.0)	18.5(18.8)	20.8(21.4)
	ΔG_{298}	0.0	5.3	17.6	19.2
B3LYP	E	-562.77452	-562.76367	-562.74704	-562.74618
	ΔE(ΔH)	0.0(0.0)	6.8(7.8)	17.2(17.6)	17.8(18.3)
	ΔG_{298}	0.0	6.1	16.4	16.1
$(C_4H_6)_3Fe$ (B3LYP*)	ΔE	9.8	31.0	0.0	0.9

Table S4. Total energies with ZPVE corrections (E in hartree), relative energies with ZPVE corrections (ΔE in kcal/mol), relative thermal Energies(ΔH in kcal/mol), and relative free energies (ΔG_{298} in kcal/mol) for the $(C_4H_6)_3Tc$ structures; comparison with the $(C_4H_6)_3Mn$ system.Error! Bookmark not defined..

		**Tc-1D** (C_1)	**Tc-2D** (C_1)	**Tc-3D** (C_1)
M06-L	E	-548.63293	-548.63130	-548.61490
	ΔE(ΔH)	0.0(0.0)	1.0(0.8)	11.3(10.4)
	ΔG_{298}	0.0	1.3	12.8
BP86	E	-548.76092	-548.76018	-548.73899
	ΔE(ΔH)	0.0(0.0)	0.5(0.3)	13.8(12.4)
	ΔG_{298}	0.0	0.6	15.5
B3LYP*	E	-548.31752	-548. 31544	-548. 29644
	ΔE(ΔH)	0.0(0.0)	1.3(1.1)	13.2(11.9)
	ΔG_{298}	0.0	1.4	14.8
B3LYP	E	-548.66419	-548.66154	-548.64436
	ΔE(ΔH)	0.0(0.0)	1.7(1.5)	12.4(11.2)

	ΔG298	0.0	1.8	14.0
$(C_4H_6)_3Mn$ (B3LYP *)	ΔE	23.9	25.9	27.5

Table S5. Total energies with ZPVE corrections (E in hartree), relative energies with ZPVE corrections (ΔE in kcal/mol), relative thermal Energies(ΔH in kcal/mol), and relative free energies (ΔG_{298} in kcal/mol) for the $(C_4H_6)_3Mo$ structures; comparison with the $(C_4H_6)_3Cr$ system.Error! Bookmark not defined..

		**Mo-1S**(C_{3h})	**Mo-2S**(C_1)	**Mo-3S**(C_1)	**Mo-1T**(C_1)	**Mo-2T**(C_1)	**Mo-3T**(C_s)
M06-L	E	-536.04474	-536.02426	-536.02360	-536.01838	-535.99805	-535.98708
	ΔE(ΔH)	0.0(0.0)	12.8(13.5)	13.3(13.5)	16.5(17.0)	29.3(30.5)	36.2(36.8)
	ΔG_{298}	0.0	10.9	12.2	14.2	25.9	34.3
BP86	E	-536.16875	-536.15267	-536.14640	-536.14416	-536.12279	-536.11094
	ΔE(ΔH)	0.0(0.0)	10.1(10.7)	14.0(14.3)	15.4(15.8)	28.8(30.0)	36.3(36.2)
	ΔG_{298}	0.0	8.1	12.9	13.1	25.4	34.3
B3LYP*	E	-535.72640	-535.71606	-535.70496	-535.70945	-535. 68920	-535. 67712
	ΔE(ΔH)	0.0(0.0)	6.5(7.1)	13.5(13.8)	10.6(11.0)	23.3(24.6)	30.9(30.8)
	ΔG_{298}	0.0	4.5	12.3	8.3	19.8	29.0
B3LYP	E	-536.06933	-536.06069	-536.04820	-536.05620	-536.03568	-536.02478
	ΔE(ΔH)	0.0(0.0)	5.4(6.0)	13.3(13.6)	8.2(8.6)	21.1(22.4)	28.0(27.9)
	ΔG_{298}	0.0	3.4	12.1	5.9	17.5	26.0
$(C_4H_6)_3Cr$ (B3LYP*)	ΔE	–	28.5	39.5	2.3	15.3	17.4

Table S6. Total energies with ZPVE corrections (E in hartree), relative energies with ZPVE corrections (ΔE in kcal/mol), relative thermal Energies(ΔH in kcal/mol), and relative free energies (ΔG_{298} in kcal/mol) for the $(C_4H_6)_3Nb$ structures; comparison with the $(C_4H_6)_3V$ system.

		**Nb-1D**(C_1)	**Nb-2D**(C_1)
M06-L	E	-524.79243	-524.77533
	ΔE(ΔH)	0.0(0.0)	10.7(9.8)
	ΔG_{298}	0.0	11.5
BP86	E	-524.91119	-524.89914
	ΔE(ΔH)	0.0(0.0)	7.6(6.7)
	ΔG_{298}	0.0	8.1
B3LYP*	E	-524.48510	-524.47609
	ΔE(ΔH)	0.0(0.0)	8.8(4.8)
	ΔG_{298}	0.0	6.3
B3LYP	E	-524.82997	-524.82288

	$\Delta E(\Delta H)$	0.0(0.0)	4.4(3.5)
	ΔG_{298}	0	5.1
$(C_4H_6)_3V$ (B3LYP)	ΔE	9.8	4.4

Table S7. Total energies with ZPVE correction (E in hartree), relative energies with ZPVE correction (ΔE in kcal/mol), relative thermal Energies(ΔH in kcal/mol), and relative free energies (ΔG_{298} in kcal/mol) for the $(C_4H_6)_3Zr$ structures.

		**Zr-1S** (C_1)	**Zr-2S** (C_1)
M06-L	E	-514.89112	-514.87692
	$\Delta E(\Delta H)$	0.0(0.0)	8.9(9.4)
	ΔG_{298}	0.0	8.5
BP86	E	-515.00107	-514.98811
	$\Delta E(\Delta H)$	0.0(0.0)	8.1(8.5)
	ΔG_{298}	0.0	8.1
B3LYP*	E	-514.59519	-514.57747
	$\Delta E(\Delta H)$	0.0(0.0)	11.1(11.6)
	ΔG_{298}	0.0	10.4
B3LYP	E	-514.94296	-514.92319
	$\Delta E(\Delta H)$	0.0(0.0)	12.4(12.8)
	ΔG_{298}	0.0	12.1
$(C_4H_6)_3Ti$ (B3LYP)	ΔE	0.0	15.5

Table S8. Our Theoretical Structural Parameters of Mo(bd)3 Compared with Those from Kaupp *et al.*[14]

	The present work		The work from Kaupp *et al.* [14]			
	M06L	BP86	HF	MP2	BP86	B3LYP
Mo-C1	2.270	2.291	2.314	2.262	2.294	2.302
Mo-C2	2.340	2.364	2.349	2.354	2.360	2.367
C1-C2	1.430	1.443	1.408	1.441	1.434	1.427
C2-C2A	1.402	1.414	1.395	1.398	1.408	1.401
C1-H1	1.092	1.100	1.076	1.087	1.094	1.087
C1-H2	1.088	1.097	1.077	1.091	1.098	1.089
C2-H3	1.088	1.098	1.074	1.087	1.094	1.086
C1-C2-C2A	118.9	119.1	120.3	119.0	119.4	119.7
H1-C1-C2	117.2	117.6	118.4	117.1	117.7	118.0
H2-C1-C2	117.5	117.1	117.3	116.9	117.3	117.2
H3-C2-C2A	119.0	118.9	118.3	119.0	118.7	118.5
$\Sigma\angle(C1)^b$	348.6	348.6	349.7	348.3	348.3	348.8

[a] Distances in Å, angles in deg. [b] Sum of angles around C1.

Table S9. The optimized geometry for $(C_4H_6)_3Pd$ structure **Pd-1S** (C_1).

M06-L	C	1.19226300	-1.00921800	-1.75565900
	C	2.61100400	0.86129900	0.50751100
	C	2.15143200	-0.58358600	0.53311300
	C	1.90256700	-1.41030300	-0.59319000
	H	2.00343000	-2.48527500	-0.43142200
	H	2.51899400	-1.12850100	1.40260200
	H	2.77030300	1.18035100	1.54583900
	H	3.60839100	0.87815700	0.04200100
	H	1.28984400	-0.00617400	-2.16524100
	H	0.94395400	-1.77224300	-2.48898600
	C	-1.33281900	-0.53986700	1.83223400
	C	-2.49919400	0.86568600	-0.74462500
	C	-2.15017200	-0.59775500	-0.51431100
	C	-1.99421900	-1.19261300	0.76610400
	H	-3.08139100	1.22783200	0.11637700
	H	-3.17976200	0.90479200	-1.60253300
	C	1.74745000	1.93379900	-0.18055100
	C	-1.35723200	1.87438700	-0.99620600
	C	-0.72789900	2.36469300	0.27085100
	C	0.56620300	2.36906900	0.62518500
	H	0.80865300	2.75078900	1.62076900
	H	-1.44271400	2.75463700	1.00168200
	H	-1.78852200	2.73801700	-1.52372700
	H	-0.61313400	1.43489300	-1.67264900
	H	1.44011100	1.61439200	-1.18022600
	H	2.39108600	2.81192500	-0.33520900
	Pd	-0.02527300	-0.83541900	0.06404900
	H	-2.49522700	-1.27425100	-1.29518600
	H	-1.37770800	0.54425400	1.92378900
	H	-1.14989800	-1.08650700	2.75298900
	H	-2.14014400	-2.27136500	0.84016200

BP86	C	1.21056600	-0.99384100	-1.78703600
	C	2.58307000	0.93639100	0.52995600
	C	2.16451200	-0.53818200	0.52565300
	C	1.94385000	-1.37750000	-0.61549200
	H	2.10167400	-2.45742300	-0.46334400
	H	2.54704400	-1.08317100	1.40130100
	H	2.70136100	1.24474700	1.58731100
	H	3.60265600	0.97950800	0.08952200
	H	1.26735900	0.02238800	-2.19378900
	H	0.97931700	-1.77273600	-2.52377400
	C	-1.26041600	-0.59883500	1.86482000
	C	-2.54827400	0.83104100	-0.72397800
	C	-2.15296000	-0.63684500	-0.48870200
	C	-1.95757300	-1.24376400	0.79738000
	H	-3.14654600	1.17839300	0.14344800
	H	-3.23391200	0.84036500	-1.59253400
	C	1.71564300	2.01838100	-0.18194100
	C	-1.43611800	1.89565300	-0.99560500
	C	-0.79761800	2.43113200	0.26520900
	C	0.51293400	2.46561700	0.61380100
	H	0.75498700	2.89160800	1.60254300
	H	-1.51617400	2.84583700	0.99331600
	H	-1.92208800	2.74599900	-1.52030400
	H	-0.68447600	1.47686400	-1.68769900
	H	1.42436800	1.69095900	-1.19294800
	H	2.37084700	2.90401600	-0.32289700
	Pd	-0.00364800	-0.87618600	0.05445500
	H	-2.50714800	-1.32720300	-1.26694600
	H	-1.27876300	0.49218400	1.97089100
	H	-1.04175100	-1.17226900	2.77323600
	H	-2.11666100	-2.33101200	0.87333800

Table S10. The optimized geometry for $(C_4H_6)_3Pd$ structure **Pd-2S** (C_1).

M06-L	C	-0.65562300	-2.16415800	0.83489000
	C	-2.73874900	0.19465600	0.67014100
	C	-2.39270700	-0.83569800	-0.38411000
	C	-1.61314100	-1.98514300	-0.21494300
	H	-1.55541700	-2.65955000	-1.06949300
	H	-2.92350400	-0.74613600	-1.33283300
	H	-3.82029000	0.19199800	0.86881900
	H	-2.25263500	-0.08059300	1.61305900
	H	-0.87696300	-1.80683400	1.84169800
	H	-0.03055900	-3.05334400	0.79946200
	C	3.73443000	-0.87415200	-0.64456200
	C	2.18100300	1.42842100	0.59796800
	C	1.77626800	-0.03609100	0.72576400
	C	2.69361300	-1.04943200	0.19802600
	H	2.76091900	1.57171300	-0.32468900
	H	2.83789700	1.74112800	1.42331600
	C	-2.25682300	1.60567000	0.24661900
	C	0.93386400	2.30320500	0.50253000
	C	0.16908000	1.83156000	-0.70254100
	C	-1.17540100	1.52357600	-0.81843300
	H	-1.54442300	1.37012000	-1.83323200
	H	0.73767000	1.86602000	-1.63505900
	H	1.19618500	3.36654000	0.39724300
	H	0.32545600	2.20896300	1.41290000
	H	-1.89501800	2.15479400	1.12254400
	H	-3.09074900	2.19356600	-0.15890700
	Pd	-0.13312600	-0.34817100	-0.20590800
	H	1.48805300	-0.27285600	1.76007300
	H	4.02128700	0.10552900	-1.02280700
	H	4.32693400	-1.71840700	-0.98477300
	H	2.48407200	-2.07128800	0.52637100

BP86	C	-0.62894800	-2.15012700	0.88346700
	C	-2.86624800	0.14998100	0.59790000
	C	-2.37199100	-0.87344600	-0.42887000
	C	-1.56004800	-2.01344400	-0.20935000
	H	-1.45115800	-2.70723600	-1.05651100
	H	-2.86145000	-0.82576400	-1.41358400
	H	-3.97554000	0.15907500	0.60371700
	H	-2.55037800	-0.15589400	1.61089500
	H	-0.87918100	-1.76757600	1.88287500
	H	0.03505300	-3.02347000	0.87554000
	C	3.77307300	-0.90041300	-0.65063900
	C	2.20975400	1.45412900	0.57684800
	C	1.78543200	-0.02262500	0.71352300
	C	2.71331500	-1.05637600	0.19371400
	H	2.78557000	1.59455500	-0.35876900
	H	2.87720900	1.76333500	1.40830700
	C	-2.29859500	1.58162900	0.28497500
	C	0.95322300	2.34500200	0.49288300
	C	0.15342200	1.83540500	-0.69315400
	C	-1.20734000	1.50674800	-0.79062100
	H	-1.58959000	1.37678900	-1.81466200
	H	0.69793000	1.89047000	-1.65052300
	H	1.22514300	3.41140100	0.34210500
	H	0.36812400	2.27900400	1.42988200
	H	-1.90161200	2.03603000	1.20913400
	H	-3.10239500	2.25288000	-0.07946400
	Pd	-0.13188500	-0.33491100	-0.19791700
	H	1.51706900	-0.24812000	1.76565500
	H	4.06949000	0.07641500	-1.05266800
	H	4.37057000	-1.76424200	-0.96523000
	H	2.50156800	-2.08052900	0.54341000

Table S11. The optimized geometry for $(C_4H_6)_3Pd$ structure **Pd-3S** (C_1).

M06-L	C	-1.12655800	-2.13335200	0.03569200
	C	-1.99736100	0.50594500	1.52777400
	C	-2.48999800	-0.04518300	0.20579600
	C	-2.23434100	-1.30771100	-0.33845000
	H	-2.76093000	-1.55169600	-1.26146300
	H	-3.23933800	0.55610300	-0.31106100
	H	-2.85081900	0.72168200	2.18604600
	H	-1.39822500	-0.25634000	2.03890400
	H	-0.81426600	-2.18050900	1.07979600
	H	-0.96408500	-3.04985200	-0.52710700
	C	2.54729300	-1.11340000	1.82663100
	C	2.60518000	0.52632300	-0.66061900
	C	1.77533200	-0.75143600	-0.56058000
	C	1.94868100	-1.51540500	0.68362900
	H	1.54052500	-2.52908300	0.66590500
	H	1.89343400	-1.40431000	-1.43377300
	H	2.75017900	0.79096600	-1.71691400
	H	3.60984900	0.38441600	-0.23060500
	H	2.99833000	-0.12821300	1.93278600
	H	2.61691300	-1.77388900	2.68641400
	C	-1.12654800	1.77615900	1.31780100
	C	1.87587100	1.68081000	0.02496900
	C	0.56181600	1.86678700	-0.67460500
	C	-0.71019400	1.91698300	-0.13422900
	H	-1.50247900	2.24522000	-0.80843000
	H	0.64282700	2.13012900	-1.73224500
	H	2.46146600	2.61045100	-0.02209700
	H	1.71730500	1.44988700	1.08604500
	H	-0.24468900	1.72596400	1.96541700
	H	-1.67671700	2.67961600	1.61344700
	Pd	-0.31639800	-0.23608000	-0.58117200

BP86	C	-0.97849500	-2.20156400	0.03253700
	C	-2.16263300	0.41455000	1.51574700
	C	-2.51295900	-0.19465300	0.15529600
	C	-2.13905200	-1.44937400	-0.37977800
	H	-2.61615400	-1.73225200	-1.33042000
	H	-3.28821400	0.34281000	-0.41221900
	H	-3.09623700	0.62411600	2.07737900
	H	-1.58631800	-0.31065000	2.11580200
	H	-0.68376100	-2.23861600	1.09020400
	H	-0.72604800	-3.09728500	-0.54927700
	C	2.75231200	-1.10553500	1.77895200
	C	2.58771200	0.70256600	-0.64472300
	C	1.81472700	-0.63266700	-0.56283700
	C	2.10441300	-1.46668700	0.63435400
	H	1.75863200	-2.51105600	0.56683200
	H	1.93924800	-1.24103600	-1.47952600
	H	2.71498500	0.98638200	-1.70852600
	H	3.60630600	0.60754500	-0.21296400
	H	3.14286800	-0.09299200	1.94055400
	H	2.91468200	-1.83056900	2.58565600
	C	-1.31382400	1.72757300	1.33620900
	C	1.78670800	1.82045900	0.05701900
	C	0.44499800	1.89822200	-0.64582500
	C	-0.85405600	1.86568400	-0.11965200
	H	-1.64925500	2.17066100	-0.81727900
	H	0.51538000	2.17987300	-1.71004600
	H	2.30786100	2.79831300	-0.01735300
	H	1.66102900	1.58689000	1.13004800
	H	-0.44598600	1.70263900	2.01744800
	H	-1.91003400	2.62307700	1.60515400
	Pd	-0.29865400	-0.23563700	-0.56163600

Table S12. The optimized geometry for $(C_4H_6)_3Pd$ structure **Pd-4S** (C_1).

M06-L	C	-1.87337100	-1.74514500	0.77069400
	C	-2.32233900	1.38086600	0.69196000
	C	-2.58637800	0.35706900	-0.38739000
	C	-2.56271900	-1.03441400	-0.26499400
	H	-2.87618500	-1.60203800	-1.14134600
	H	-2.96469700	0.74957500	-1.33227400
	H	-3.23039500	1.96758900	0.89356500
	H	-2.07617300	0.86537300	1.62735000
	H	-1.87132200	-1.36031900	1.79172200
	H	-1.83605200	-2.82971100	0.70051000
	C	1.25875100	-1.52239100	0.64490000
	C	3.30288400	0.80016000	0.43819000
	C	3.33236200	-0.48799700	-0.33758100
	C	2.41543200	-1.47921400	-0.24941700
	H	2.54026000	-2.32931500	-0.92399300
	H	4.14868800	-0.60686400	-1.04913000
	H	4.00095300	1.49886200	-0.03818100
	H	3.69778500	0.64906800	1.45413200
	H	1.41734200	-0.97854900	1.58596800
	H	0.93833700	-2.54354100	0.86738800
	C	-1.14529900	2.30584800	0.29451900
	C	1.92792900	1.49489400	0.55682500
	C	1.04557800	1.28837700	-0.64456200
	C	-0.27136100	1.66628000	-0.76993300
	H	-0.68204100	1.68159800	-1.77996500
	H	1.56159800	0.98168100	-1.55588100
	H	2.10240000	2.57346300	0.68517100
	H	1.40522800	1.17780100	1.46866200
	H	-0.54985800	2.55479200	1.17965000
	H	-1.52017900	3.26040400	-0.09896000
	Pd	-0.42031500	-0.51860600	-0.19116600

BP86	C	-1.82311000	-1.78956300	0.79782500
	C	-2.42206600	1.38340400	0.66665300
	C	-2.60050500	0.30084200	-0.39657200
	C	-2.53840600	-1.10513100	-0.25210600
	H	-2.83400100	-1.69782600	-1.13110700
	H	-2.98733900	0.65598900	-1.36429200
	H	-3.35915500	1.96965200	0.76823500
	H	-2.23180400	0.91702100	1.64920800
	H	-1.81979600	-1.39722600	1.82501200
	H	-1.73882600	-2.88125400	0.72306200
	C	1.26978200	-1.52089600	0.62275600
	C	3.33241800	0.85422000	0.43011200
	C	3.38540200	-0.46359000	-0.32393200
	C	2.46393000	-1.46957700	-0.25053700
	H	2.61551100	-2.33303400	-0.91920500
	H	4.23301700	-0.59199700	-1.01310100
	H	4.01137500	1.56121600	-0.08396900
	H	3.74984800	0.73450400	1.45266100
	H	1.41871800	-1.00061000	1.58821600
	H	0.95162400	-2.55900600	0.81519400
	C	-1.21681700	2.31289300	0.29099400
	C	1.92889700	1.53038500	0.56024600
	C	1.01687300	1.26211200	-0.62688400
	C	-0.32308300	1.63963800	-0.76185500
	H	-0.71284300	1.67933500	-1.79010200
	H	1.53723200	0.97458000	-1.55256900
	H	2.09179900	2.62558700	0.64095200
	H	1.43703700	1.22722800	1.50215600
	H	-0.63446400	2.55798400	1.19667400
	H	-1.57713900	3.27574000	-0.12478300
	Pd	-0.41284100	-0.50709400	-0.18961800

Table S13. The optimized geometry for the $(C_4H_6)_3Rh$ structure **Rh-1D** (C_1).

M06-L	C	1.17200600	-1.73742000	-1.19148500
	C	2.63292900	0.87677500	-0.37355100
	C	2.22435700	-0.30122400	0.50262900
	C	1.85819300	-1.59508100	0.04740400
	H	1.87363800	-2.42344600	0.75463000
	H	2.55578100	-0.24016800	1.54053500
	H	3.64640800	1.20404000	-0.10442400
	H	2.68846500	0.55887700	-1.42097700
	H	1.44647400	-1.12061300	-2.04951200
	H	0.76753100	-2.71492700	-1.45063600
	C	-1.74234300	-1.38358200	1.39831100
	C	-2.63960600	0.82932900	-0.65856500
	C	-1.88014800	-0.45888100	-0.92333600
	C	-1.78465700	-1.54092800	0.00501500
	H	-3.23518800	0.72069000	0.26062000
	H	-3.35291300	1.03260000	-1.46840000
	C	1.61125800	2.03784300	-0.23442800
	C	-1.66615200	1.99158800	-0.47222000
	C	-0.79426100	1.65227100	0.70720300
	C	0.58776800	1.68872800	0.82420000
	H	0.98334700	1.62458000	1.83842900
	H	-1.34437000	1.54477600	1.64550500
	H	-2.19976800	2.93886300	-0.29536700
	H	-1.06399100	2.12502800	-1.38068500
	H	1.10832800	2.20843800	-1.19355500
	H	2.10961400	2.98392500	0.02403700
	H	-1.80016700	-0.74170700	-1.97441200
	H	-2.13560400	-0.49034800	1.87764300
	H	-1.57000900	-2.24476300	2.03715500
	H	-1.56231800	-2.53094600	-0.39612200
	Rh	0.08019300	-0.37314200	0.08818800

BP86	C	1.14234700	-1.68278800	-1.28974900
	C	2.71116700	0.85501200	-0.32514100
	C	2.22250200	-0.35584100	0.49819100
	C	1.82303500	-1.62950300	-0.02867300
	H	1.82303600	-2.50731900	0.63271500
	H	2.55184500	-0.35952800	1.54845900
	H	3.70888600	1.16585900	0.04395700
	H	2.84818900	0.56385800	-1.38225200
	H	1.42495800	-1.00757500	-2.11065300
	H	0.70342900	-2.63783300	-1.60859900
	C	-1.63567000	-1.36948400	1.45448100
	C	-2.68470700	0.83771400	-0.65391800
	C	-1.92287300	-0.47262900	-0.89652600
	C	-1.77921500	-1.55190600	0.05143700
	H	-3.28925500	0.75205100	0.27186600
	H	-3.39503700	1.03033500	-1.48292200
	C	1.68181000	2.03621900	-0.23120100
	C	-1.68902400	2.00866300	-0.49184000
	C	-0.78669300	1.64934800	0.68003500
	C	0.61562600	1.67675800	0.80356500
	H	0.99336200	1.63734100	1.83689100
	H	-1.33146800	1.57911100	1.63604600
	H	-2.22162900	2.96415200	-0.29071600
	H	-1.10824300	2.14050600	-1.42374300
	H	1.21511200	2.20898800	-1.21726300
	H	2.18132900	2.98563600	0.05769800
	H	-1.86262900	-0.77895100	-1.95187300
	H	-2.05315700	-0.49711500	1.96816800
	H	-1.39749500	-2.23671200	2.08203800
	H	-1.57987100	-2.55724300	-0.34995800
	Rh	0.05775200	-0.36566500	0.09591500

Table S14. The optimized geometry for the $(C_4H_6)_3Rh$ structure **Rh-2D** (C_1).

M06-L	C	-1.64089100	-1.89986700	0.11091500
	C	-2.00260200	1.20718600	1.07401900
	C	-2.25675100	0.50062700	-0.24223700
	C	-2.32251700	-0.86655200	-0.58360700
	H	-2.68734400	-1.08415700	-1.58842300
	H	-2.64654700	1.15902600	-1.02128600
	H	-2.88885800	1.78091300	1.38460700
	H	-1.79493500	0.47564500	1.86275000
	H	-1.58453700	-1.88269600	1.20004100
	H	-1.65705700	-2.89573400	-0.32977800
	C	0.82391600	-0.82901100	2.01753400
	C	2.93557700	-0.08108600	-0.21782200
	C	1.88128000	-1.17288200	-0.26063800
	C	1.14577700	-1.58615000	0.88469800
	H	0.74419900	-2.59889900	0.85504600
	H	2.07781400	-1.96792500	-0.98082100
	H	3.42539400	-0.04233900	-1.19926500
	H	3.72687000	-0.32472300	0.50844400
	H	1.27941700	0.12946800	2.23777300
	H	0.17461000	-1.24938800	2.78186800
	C	-0.76426700	2.13638300	0.88717000
	C	2.33101200	1.29171700	0.06442300
	C	1.12983100	1.45840800	-0.81948700
	C	-0.15429300	1.86392200	-0.47374400
	H	-0.76373100	2.21327300	-1.30834800
	H	1.36143700	1.48692000	-1.88713700
	H	3.06451500	2.08534500	-0.14029300
	H	2.05127400	1.40207900	1.11645300
	H	-0.03449800	1.94165300	1.68104700
	H	-1.04403500	3.19687000	0.96631900
	Rh	-0.20978700	-0.35470000	-0.46191900

BP86	C	-1.58091700	-1.97411300	0.10347100
	C	-2.11505200	1.17314700	1.07158000
	C	-2.29432900	0.42760300	-0.25457200
	C	-2.29353500	-0.95436800	-0.60482900
	H	-2.63753300	-1.18561600	-1.62501000
	H	-2.70929800	1.06362200	-1.05269000
	H	-3.03958600	1.73304500	1.32660100
	H	-1.91425000	0.46344400	1.89155900
	H	-1.54766300	-1.97004600	1.20115400
	H	-1.53806100	-2.96893900	-0.36146200
	C	0.97045300	-0.84812900	2.07418500
	C	2.95667800	-0.00188900	-0.27252800
	C	1.90127900	-1.11486400	-0.29851900
	C	1.20894700	-1.56373700	0.87889800
	H	0.83844200	-2.59833900	0.85501500
	H	2.08016400	-1.89990700	-1.04872600
	H	3.41960900	0.05597300	-1.27687300
	H	3.77441700	-0.24439400	0.43962100
	H	1.42200200	0.12370700	2.28739600
	H	0.37497000	-1.30598400	2.87271000
	C	-0.88508000	2.14891600	0.90570400
	C	2.32293800	1.36880700	0.04615200
	C	1.07687800	1.48976200	-0.80909000
	C	-0.23432600	1.85960000	-0.45222000
	H	-0.84216500	2.21129800	-1.30034100
	H	1.29220600	1.54016500	-1.88991700
	H	3.03024500	2.19107500	-0.19094000
	H	2.07500500	1.46190300	1.11630500
	H	-0.17148400	1.98792300	1.73149900
	H	-1.20623200	3.21052500	0.94740800
	Rh	-0.19787500	-0.35408600	-0.45006000

Table S15. The optimized geometry for the $(C_4H_6)_3Rh$ structure **Rh-3D** (C_1).

M06-L	C	1.08703900	-1.61284400	0.90665300
	C	3.78579300	-0.10422000	0.63471200
	C	3.03009400	-0.56207400	-0.38623100
	C	1.80514200	-1.33861900	-0.28164700
	H	1.56597600	-1.92974300	-1.16520700
	H	3.36687700	-0.36011200	-1.40504600
	H	4.69321600	0.46410100	0.45415000
	H	3.51653200	-0.29014100	1.67333500
	H	1.49542300	-1.29594000	1.86611100
	H	0.47805500	-2.51688900	0.94591800
	C	0.16990000	1.53146900	-1.64238500
	C	0.95173300	1.69470800	1.10161200
	C	-0.36180800	2.01478600	0.72312900
	C	-0.76621900	1.91944300	-0.65712800
	H	-1.82010300	2.03308700	-0.90197200
	H	-1.14168700	2.12804500	1.47563300
	H	1.18664300	1.60758000	2.16002900
	H	1.80086200	1.91949800	0.45834200
	H	1.21991400	1.81152100	-1.56842900
	H	-0.18555800	1.32854400	-2.64970600
	C	-1.09326000	-1.41289500	-1.25145500
	C	-3.85641600	-0.31540000	1.07322500
	C	-3.02107800	-0.38825100	0.01375700
	C	-1.85993600	-1.24616800	-0.07394600
	H	-1.73146300	-1.95810700	0.74403500
	H	-3.23299300	0.22233900	-0.86887800
	H	-4.72664300	0.33339900	1.06924000
	H	-3.68436900	-0.91182100	1.96750100
	H	-1.44013500	-0.95357600	-2.17802600
	H	-0.53224600	-2.33757600	-1.38715100
	Rh	-0.00120500	-0.00830700	-0.03670300

BP86	C	1.07297600	-1.62369000	0.87520300
	C	3.89454600	-0.22488400	0.64025500
	C	3.07010500	-0.56334000	-0.39162700
	C	1.80393000	-1.30542500	-0.31330200
	H	1.57156400	-1.88047100	-1.22111100
	H	3.38802700	-0.29553600	-1.41241400
	H	4.83042600	0.31702100	0.46090600
	H	3.66208200	-0.49362800	1.67841800
	H	1.47358900	-1.33169300	1.85489500
	H	0.46863200	-2.54291300	0.88286000
	C	0.21904900	1.56763500	-1.62070400
	C	0.94833700	1.67142100	1.17749900
	C	-0.35855500	2.03281900	0.76310500
	C	-0.73546900	1.97412800	-0.63874100
	H	-1.78719700	2.13051100	-0.90820700
	H	-1.16000200	2.15921700	1.50494500
	H	1.14029400	1.54839300	2.25064900
	H	1.83127600	1.89018700	0.56553100
	H	1.28011100	1.83294300	-1.53277000
	H	-0.13200600	1.38122500	-2.64317000
	C	-1.11172300	-1.34758900	-1.31097200
	C	-3.93401200	-0.41552100	1.07131200
	C	-3.06994500	-0.39519400	0.01397600
	C	-1.88011400	-1.22973800	-0.11101100
	H	-1.75220400	-1.98953900	0.67537600
	H	-3.29178100	0.26905900	-0.83775400
	H	-4.83054800	0.21441300	1.08980500
	H	-3.75976500	-1.06876700	1.93665800
	H	-1.46468000	-0.84592300	-2.22314700
	H	-0.55626500	-2.27862300	-1.49064500
	Rh	-0.00947300	0.00312000	-0.03468400

Table S16. The optimized geometry for the $(C_4H_6)_3Rh$ structure **Rh-4D** (C_1).

M06-L	C	2.10214600	-0.65308400	1.40285400
	C	1.72984300	-1.11381700	-1.34903700
	C	2.35214400	0.08765900	-0.92671600
	C	2.55929400	0.31475400	0.47750400
	H	2.87837900	1.30274600	0.80653700
	H	2.55326800	0.89272300	-1.63139500
	H	1.42863000	-1.20559200	-2.39029000
	H	1.92759600	-2.05736100	-0.84199400
	H	2.22372900	-1.71455100	1.18491000
	H	2.11357900	-0.40217500	2.46155200
	C	-2.10946500	2.75834400	0.53473800
	C	-1.01055700	0.62664200	-1.40255700
	C	-0.35528500	1.79546500	-0.97073300
	C	-0.84330100	2.69791000	0.05825600
	H	-0.11385900	3.41150900	0.44570500
	H	0.49110700	2.15968800	-1.55463400
	H	-0.77001900	0.19983500	-2.37453700
	H	-2.01606300	0.39955100	-1.04732400
	H	-2.89496300	2.10969200	0.14873800
	H	-2.38710400	3.46111700	1.31439900
	C	-1.19798100	-0.42313700	1.45892100
	C	-2.99195800	-2.00844900	-0.49108000
	C	-1.70875400	-2.29251300	-0.19538400
	C	-0.90463600	-1.65063900	0.83784700
	H	-0.16026100	-2.29351900	1.30932700
	H	-1.21355300	-3.09393000	-0.74712700
	H	-3.52114300	-2.53410500	-1.27994700
	H	-3.54805500	-1.24901100	0.05724000
	H	-2.09953700	0.12505200	1.18325100
	H	-0.82044400	-0.21789900	2.45850000
	Rh	0.44888400	-0.00285800	0.08643100

BP86	C	2.13080700	-0.66456800	1.42261900
	C	1.76534100	-1.08621300	-1.37803200
	C	2.38845900	0.11926500	-0.92060200
	C	2.58731000	0.32470800	0.50087600
	H	2.90408400	1.31789500	0.84914700
	H	2.60418700	0.94219700	-1.61560200
	H	1.46920700	-1.14857800	-2.43281300
	H	1.96979700	-2.05132600	-0.89716800
	H	2.26004400	-1.73264800	1.20004700
	H	2.12312400	-0.41192000	2.49100100
	C	-2.21104700	2.81319700	0.48882400
	C	-0.97059200	0.64829300	-1.42667000
	C	-0.34655900	1.81990300	-0.91356700
	C	-0.90139600	2.71622500	0.10800000
	H	-0.17840100	3.40687200	0.57117700
	H	0.51831500	2.22632200	-1.45978600
	H	-0.68050900	0.27189600	-2.41607300
	H	-1.98973100	0.38119000	-1.12154300
	H	-2.99395900	2.20276900	0.02167800
	H	-2.52303300	3.52230300	1.26421400
	C	-1.16406000	-0.45334900	1.46429700
	C	-3.07959300	-2.05941600	-0.44030800
	C	-1.75823400	-2.30853600	-0.22737900
	C	-0.90081100	-1.67833100	0.79000900
	H	-0.15403000	-2.35389000	1.23388100
	H	-1.27080800	-3.08506500	-0.83831800
	H	-3.63704400	-2.59092100	-1.22046500
	H	-3.63693500	-1.34037000	0.17340100
	H	-2.06602200	0.12618100	1.22859700
	H	-0.76087400	-0.29290100	2.47275700
	Rh	0.46232900	-0.01193500	0.08194400

Table S17. The optimized geometry for the $(C_4H_6)_2Ru$ structure **Ru-1S** (C_1).

M06-L	C	1.38228600	-1.48093600	-1.22604600
	C	2.51659600	1.23752200	-0.26336000
	C	2.25483500	-0.02660900	0.56100300
	C	2.01570100	-1.32693000	0.04312200
	H	2.08809300	-2.17748500	0.72183100
	H	2.56700200	0.02145000	1.60405500
	H	3.19241300	1.91093700	0.27713500
	H	3.00783700	1.00326400	-1.21651900
	H	1.66771200	-0.82070100	-2.05217100
	H	1.11143200	-2.48539400	-1.54991000
	C	-0.95299700	-1.43745900	1.42746000
	C	-2.85315000	0.32907300	-0.46692600
	C	-1.87546300	-0.78785300	-0.80662500
	C	-1.37257100	-1.77213500	0.08790800
	H	-3.31217900	0.12737000	0.51250700
	H	-3.67303900	0.36313200	-1.19674500
	C	1.15333800	1.90467700	-0.50663000
	C	-2.10595200	1.65789200	-0.38360300
	C	-1.08134700	1.46989600	0.70879100
	C	0.30032400	1.74287100	0.73628100
	H	0.79054300	1.93382200	1.68970900
	H	-1.53674000	1.36939400	1.69892700
	H	-2.78120800	2.49859900	-0.15534700
	H	-1.63089500	1.88036000	-1.34883600
	H	0.63907100	1.35058500	-1.33752900
	H	1.19801200	2.93003400	-0.90410700
	H	-1.88859900	-1.09800800	-1.85411100
	H	-1.58507600	-0.80721600	2.05763900
	H	-0.43231500	-2.22003200	1.98264400
	H	-1.01426400	-2.71163600	-0.33655900
	Ru	0.12051400	-0.27564900	0.04407100

BP86	C	1.26284800	-1.58759800	-1.25301400
	C	2.59451300	1.13383800	-0.33401000
	C	2.24898800	-0.12996200	0.49104700
	C	1.97218100	-1.44817100	-0.01111800
	H	2.07159400	-2.30990700	0.66523300
	H	2.58204600	-0.08885600	1.53854400
	H	3.43852400	1.66212400	0.14831800
	H	2.93344300	0.86790000	-1.35286400
	H	1.48516100	-0.90674700	-2.09510600
	H	0.94761400	-2.59146100	-1.56946500
	C	-1.05293600	-1.37872400	1.46099000
	C	-2.85028800	0.48582600	-0.53947000
	C	-1.93896000	-0.72086200	-0.81799500
	C	-1.51028100	-1.72026900	0.11421500
	H	-3.39325600	0.32894300	0.41427500
	H	-3.61749600	0.57669600	-1.33404600
	C	1.33056300	2.05944400	-0.40152100
	C	-2.00140000	1.77121700	-0.42036700
	C	-1.00306100	1.49216300	0.69685800
	C	0.40339200	1.70883500	0.77176300
	H	0.84435700	1.84588300	1.76992700
	H	-1.49450800	1.40119200	1.68103800
	H	-2.63120900	2.65655500	-0.18173700
	H	-1.49703000	1.97758500	-1.38343700
	H	0.79547700	1.88428500	-1.36015200
	H	1.59295700	3.13865300	-0.40903300
	H	-1.94266400	-1.06006800	-1.86646000
	H	-1.65338300	-0.71881300	2.10602800
	H	-0.54956200	-2.18779100	2.01403600
	H	-1.21780800	-2.70229700	-0.28723100
	Ru	0.10391800	-0.31291600	0.06722400

Table S18. The optimized geometry for the $(C_4H_6)_2Ru$ structure **Ru-2S** (C_1).

M06-L	C	1.05814000	-0.88920800	-1.41445300
	C	3.70940700	-0.19425500	0.05382500
	C	2.67038700	-0.86656400	0.58142400
	C	1.45642000	-1.29450700	-0.11182200
	H	1.07610400	-2.25256400	0.24759200
	H	2.72553200	-1.14846000	1.63547600
	H	4.57094700	0.07949600	0.65534700
	H	3.73654600	0.07902300	-1.00048500
	H	1.66145400	-0.15004300	-1.94393100
	H	0.54922400	-1.59339100	-2.07132500
	C	-2.01538900	-0.86397700	-1.18248600
	C	-1.96069600	-0.23431000	1.51794800
	C	-1.41226500	-1.47649900	1.13393200
	C	-1.45920100	-1.79098200	-0.26439100
	H	-0.89956300	-2.65606500	-0.61982000
	H	-0.83479200	-2.10050000	1.81118000
	H	-1.77345600	0.13666700	2.52273500
	H	-2.88232300	0.11909600	1.06009000
	H	-2.91738500	-0.31066100	-0.92801600
	H	-1.89359900	-1.07237100	-2.24336600
	C	-1.41687500	1.97202100	-0.31560300
	C	0.71019600	1.44171400	1.29322700
	C	0.96495300	1.74160900	-0.07473300
	C	-0.14079900	2.00131000	-0.94603100
	H	0.00273900	2.07907300	-2.02015000
	H	1.95679400	1.58379500	-0.49569200
	H	1.55808700	1.09104600	1.87946400
	H	-0.03545200	2.00067000	1.85631400
	H	-1.54670100	2.45350800	0.65314300
	H	-2.30686000	1.97380600	-0.94144400
	Ru	-0.35756800	0.05476700	-0.03823000

BP86	C	1.07592800	-0.87151900	-1.42366700
	C	3.79931100	-0.20632600	0.04471000
	C	2.72500300	-0.84181800	0.58411300
	C	1.49127700	-1.26580500	-0.10768700
	H	1.12659500	-2.23792500	0.25804900
	H	2.77301400	-1.11277000	1.65176800
	H	4.67227300	0.04774700	0.65744800
	H	3.84776200	0.04477300	-1.02302000
	H	1.66431100	-0.11952700	-1.96720400
	H	0.58026700	-1.59946100	-2.07950000
	C	-2.00958500	-0.91564300	-1.20163200
	C	-2.00081000	-0.22344200	1.51483400
	C	-1.42821600	-1.47986700	1.15818900
	C	-1.45620400	-1.82831600	-0.24574100
	H	-0.89861800	-2.71542800	-0.57671100
	H	-0.86263100	-2.09778800	1.86617600
	H	-1.82277200	0.16421700	2.52535300
	H	-2.93593500	0.10911900	1.04816100
	H	-2.92983000	-0.36227900	-0.97977300
	H	-1.85702900	-1.14354000	-2.26430200
	C	-1.44670100	1.97567100	-0.40595000
	C	0.64192100	1.47098400	1.34706400
	C	0.94967600	1.77455000	-0.02112200
	C	-0.12428500	2.01887100	-0.95657300
	H	0.08000400	2.09797900	-2.03079700
	H	1.97208500	1.64097000	-0.39534700
	H	1.47404500	1.12112800	1.97031100
	H	-0.14237700	2.01498300	1.88787200
	H	-1.65311300	2.45451500	0.55972600
	H	-2.29736200	1.96159500	-1.09889400
	Ru	-0.36578600	0.04744600	-0.03928500

Table S19. The optimized geometry for the $(C_4H_6)_3Ru$ structure **Ru-1T** (C_1).

M06-L	C	-0.84573700	-0.87150400	1.75449000
	C	-2.97156200	0.46686900	-0.12425800
	C	-2.21186100	-0.84981800	-0.31356900
	C	-1.55629500	-1.55360300	0.72958500
	H	-1.40230000	-2.62742100	0.61153800
	H	-2.59574900	-1.47994100	-1.11513000
	H	-3.54149000	0.64881100	-1.04312700
	H	-3.71243000	0.37801400	0.68209900
	H	-1.22331700	0.06070500	2.16787200
	H	-0.27261800	-1.47340900	2.45581800
	C	1.85244100	-1.20123100	-1.41144500
	C	2.47464700	1.21973700	0.64148700
	C	1.96956300	-0.19841300	0.87226700
	C	2.08025200	-1.30876000	-0.01293400
	H	3.05775100	1.26381200	-0.28784000
	H	3.15081400	1.51447800	1.45478000
	C	-2.04078100	1.66613400	0.10335300
	C	1.28491800	2.17709800	0.52513600
	C	0.48625300	1.68617600	-0.65483600
	C	-0.87595800	1.47580400	-0.83267900
	H	-1.18632300	1.33352700	-1.87056600
	H	1.06527300	1.69408700	-1.58189000
	H	1.62019800	3.21267600	0.36165900
	H	0.69256500	2.15908800	1.44616900
	H	-1.70748100	1.73033600	1.14273800
	H	-2.56333900	2.60925000	-0.11602400
	H	1.93153800	-0.47709000	1.92403700
	H	2.17524300	-0.31713500	-1.95856700
	H	1.81587500	-2.11543700	-1.99878400
	H	2.05522900	-2.30564000	0.42943400
	Ru	0.06284700	-0.50135600	-0.23554100

BP86	C	-0.82808700	-0.82547400	1.78081900
	C	-3.01279000	0.43347800	-0.11952600
	C	-2.22258300	-0.88226200	-0.29028600
	C	-1.53351400	-1.55936000	0.76895700
	H	-1.36087500	-2.64381500	0.67938500
	H	-2.59707300	-1.53925200	-1.08934600
	H	-3.58755300	0.59545500	-1.05145600
	H	-3.76145400	0.34229800	0.69419900
	H	-1.23049000	0.11657300	2.17404300
	H	-0.22531900	-1.39644200	2.49878600
	C	1.85157000	-1.25101800	-1.39887300
	C	2.50366100	1.23827100	0.62077600
	C	1.98947400	-0.18429700	0.87817100
	C	2.09019400	-1.33237600	0.01531300
	H	3.07974000	1.27032800	-0.32574300
	H	3.19505200	1.54705600	1.43095000
	C	-2.08866900	1.65859200	0.09531900
	C	1.29877100	2.20201000	0.50278900
	C	0.48039600	1.67156900	-0.66149200
	C	-0.90605800	1.44816300	-0.83406200
	H	-1.21864800	1.31540300	-1.88447800
	H	1.05338500	1.68604900	-1.60644200
	H	1.63387800	3.24492000	0.31267800
	H	0.71641500	2.19986300	1.44234300
	H	-1.76415900	1.74490600	1.14710200
	H	-2.62325500	2.60210500	-0.14811700
	H	1.93547100	-0.43764600	1.94659600
	H	2.18852600	-0.37560300	-1.97212200
	H	1.79655200	-2.18432600	-1.97393500
	H	2.05913300	-2.32673400	0.48781100
	Ru	0.06764700	-0.48783900	-0.24794700

Table S20. The optimized geometry for the $(C_4H_6)_3Ru$ structure **Ru-2T** (C_1).

M06-L	C	0.91220200	1.75458300	-1.77602300
	C	0.02489400	-1.10591700	-2.51077600
	C	-0.82128800	-0.00218200	-1.89487900
	C	-0.43308400	1.36682800	-2.04959500
	H	-1.19732300	2.14424100	-2.13629000
	H	-1.89641500	-0.18700500	-1.92081600
	H	-0.61392800	-1.67900200	-3.19757400
	H	0.80136100	-0.64851300	-3.13868800
	H	1.72332800	1.05903300	-2.00524100
	H	1.18697000	2.80428800	-1.84373800
	C	0.91220200	1.75458300	1.77602300
	C	0.02489400	-1.10591700	2.51077600
	C	-0.82128800	-0.00218200	1.89487900
	C	-0.43308400	1.36682800	2.04959500
	H	-1.19732300	2.14424100	2.13629000
	H	-1.89641500	-0.18700500	1.92081600
	H	-0.61392800	-1.67900200	3.19757400
	H	0.80136100	-0.64851300	3.13868800
	H	1.72332800	1.05903300	2.00524100
	H	1.18697000	2.80428800	1.84373800
	C	0.70625800	-2.08621100	-1.55255800
	C	0.70625800	-2.08621100	1.55255800
	C	-0.25367400	-2.81544000	0.67037900
	C	-0.25367400	-2.81544000	-0.67037900
	H	-1.02990400	-3.38553100	-1.18671000
	H	-1.02990400	-3.38553100	1.18671000
	H	1.28073400	-2.80985900	2.14928400
	H	1.44336800	-1.53869200	0.94972300
	H	1.44336800	-1.53869200	-0.94972300
	H	1.28073400	-2.80985900	-2.14928400
	Ru	-0.11409300	0.98050400	0.00000000

BP86	C	0.41125000	2.00982600	-1.74814700
	C	0.43208700	-1.07001400	-2.47729400
	C	-0.68174200	-0.22632900	-1.84840900
	C	-0.74716000	1.20813000	-2.06283800
	H	-1.71399300	1.70617600	-2.25042200
	H	-1.65770700	-0.73807100	-1.82606600
	H	-0.03940100	-1.67908800	-3.27739700
	H	1.14911900	-0.39980300	-2.99005300
	H	1.41942600	1.59470900	-1.90367200
	H	0.34791000	3.10163900	-1.83936300
	C	0.41125000	2.00982600	1.74814700
	C	0.43208700	-1.07001400	2.47729400
	C	-0.68174200	-0.22632900	1.84840900
	C	-0.74716000	1.20813000	2.06283800
	H	-1.71399300	1.70617600	2.25042200
	H	-1.65770700	-0.73807100	1.82606600
	H	-0.03940100	-1.67908800	3.27739700
	H	1.14911900	-0.39980300	2.99005300
	H	1.41942600	1.59470900	1.90367200
	H	0.34791000	3.10163900	1.83936300
	C	1.24663700	-2.01722700	-1.55702400
	C	1.24663700	-2.01722700	1.55702400
	C	0.41123600	-2.91529400	0.67807700
	C	0.41123600	-2.91529400	-0.67807700
	H	-0.25459700	-3.62174200	-1.20076200
	H	-0.25459700	-3.62174200	1.20076200
	H	1.89520300	-2.64060100	2.20865200
	H	1.92378000	-1.40362900	0.93713300
	H	1.92378000	-1.40362900	-0.93713300
	H	1.89520300	-2.64060100	-2.20865200
	Ru	-0.43197400	1.00660800	0.00000000

Table S21. The optimized geometry for the $(C_4H_6)_3Tc$ structure **Tc-1D** (C_1).

M06-L	C	1.40109200	-1.52690000	1.25158500
	C	0.46121400	-1.71400400	-1.37826400
	C	1.76246900	-1.18455300	-1.15299600
	C	2.24813100	-1.09158700	0.18510500
	H	3.17180100	-0.54680600	0.37375600
	H	2.31056400	-0.67755400	-1.94612200
	H	0.03729900	-1.60931400	-2.37572100
	H	0.14173200	-2.61184400	-0.85025900
	H	0.85405700	-2.46803500	1.15530700
	H	1.72880000	-1.31075000	2.26716800
	C	0.69997600	1.94737700	1.21201500
	C	-0.85571200	1.49553000	-1.07060300
	C	0.52696600	1.80517300	-1.22934900
	C	1.32534500	2.01448800	-0.06442200
	H	2.40948100	2.04141000	-0.16743400
	H	1.02302300	1.70105500	-2.19321100
	H	-1.41124700	1.17755700	-1.95028100
	H	-1.46352000	2.03231400	-0.33877100
	H	-0.26011200	2.43518100	1.37144700
	H	1.34094300	1.92855500	2.09165700
	C	-1.26062000	-0.10287800	1.61256600
	C	-3.75149400	0.01698900	-0.20408300
	C	-2.71995300	-0.83082100	-0.35048700
	C	-1.61010000	-1.00742500	0.59065800
	H	-1.28982100	-2.04347500	0.71515400
	H	-2.69591700	-1.47702000	-1.23131600
	H	-4.53551300	0.08993200	-0.95211800
	H	-3.83889900	0.66255200	0.66861200
	H	-1.79989200	0.84254000	1.68134200
	H	-0.84847900	-0.46296800	2.55067200
	Tc	0.36655400	0.03182200	0.06325000

BP86	C	1.39841500	-1.54618000	1.27701400
	C	0.51587200	-1.70964200	-1.42869800
	C	1.81897200	-1.18262200	-1.14465100
	C	2.27173400	-1.10358600	0.21916300
	H	3.20164100	-0.56011000	0.43504600
	H	2.40146800	-0.66795400	-1.92173700
	H	0.12590600	-1.57869300	-2.44668900
	H	0.16494800	-2.61741800	-0.92240400
	H	0.84568300	-2.49264300	1.17409000
	H	1.70902900	-1.32032200	2.30589800
	C	0.71739600	1.94715200	1.24443200
	C	-0.85173200	1.51784600	-1.09553700
	C	0.54302500	1.83537000	-1.22746500
	C	1.34103300	2.03159300	-0.04465100
	H	2.43453600	2.06996700	-0.14741800
	H	1.05388300	1.75263100	-2.19658700
	H	-1.39113500	1.19936400	-1.99549000
	H	-1.48274800	2.03543100	-0.35940900
	H	-0.24902700	2.43317000	1.42484000
	H	1.37202700	1.90540900	2.12528900
	C	-1.26350200	-0.12260000	1.60373100
	C	-3.85389700	-0.00333100	-0.16069500
	C	-2.77637800	-0.79996200	-0.37722500
	C	-1.62835000	-0.99819400	0.53746400
	H	-1.34001100	-2.05503000	0.64491500
	H	-2.75246500	-1.40286000	-1.29984800
	H	-4.66335500	0.06697200	-0.89711500
	H	-3.95887300	0.58897300	0.75728700
	H	-1.80059900	0.82978200	1.70610400
	H	-0.87450100	-0.52974300	2.54373300
	Tc	0.36762900	0.02669800	0.06167900

Table S22. The optimized geometry for the $(C_4H_6)_3Tc$ structure **Tc-2D** (C_1).

M06-L	C	0.99211200	-0.74638900	-1.49908100
	C	3.72954200	-0.46108400	-0.04231300
	C	2.63450700	-1.06490000	0.45701300
	C	1.36684500	-1.30422100	-0.23530500
	H	0.93796300	-2.27986000	0.00467800
	H	2.68351800	-1.44325900	1.48182200
	H	4.62755600	-0.33321600	0.55517100
	H	3.76050200	-0.09412300	-1.06778100
	H	1.64947000	0.00766800	-1.93547200
	H	0.50097800	-1.37012700	-2.24601300
	C	-2.19974500	-0.48024200	-1.14611100
	C	-1.83556200	-0.59445900	1.63414400
	C	-1.46679000	-1.70423100	0.84819800
	C	-1.62499800	-1.64495400	-0.56710300
	H	-1.16868600	-2.42409000	-1.17489500
	H	-0.88283300	-2.51569900	1.27766000
	H	-1.55771800	-0.57753000	2.68333600
	H	-2.70979400	0.00342600	1.37657300
	H	-3.06703800	-0.00927500	-0.68118300
	H	-2.17392100	-0.39150900	-2.23034600
	C	-1.06181500	2.16811800	-0.64977500
	C	0.37731700	1.38887200	1.61618300
	C	1.08160800	1.67809000	0.41665500
	C	0.34581800	2.09138900	-0.73068300
	H	0.85433300	2.15039600	-1.69002200
	H	2.13466400	1.43000300	0.30596400
	H	0.93894400	0.93447700	2.43054800
	H	-0.44652700	2.02548600	1.94194200
	H	-1.54242300	2.53029800	0.26002900
	H	-1.63268300	2.32061800	-1.56063800
	Tc	-0.39393800	0.09489300	-0.00796000

BP86	C	0.99520500	-0.79165700	-1.49789300
	C	3.79517100	-0.48547800	-0.05755100
	C	2.67539700	-1.05130700	0.47053600
	C	1.39026200	-1.31052700	-0.21069100
	H	0.96673200	-2.28734200	0.06936000
	H	2.72257100	-1.39271300	1.51884700
	H	4.70110500	-0.35528700	0.54608600
	H	3.84061500	-0.16340100	-1.10613000
	H	1.63927000	-0.03674500	-1.96880600
	H	0.49748200	-1.44805100	-2.22448400
	C	-2.23027300	-0.45715500	-1.15540900
	C	-1.83987100	-0.57002900	1.65442000
	C	-1.51023400	-1.70448800	0.85906600
	C	-1.68363500	-1.64615100	-0.56611000
	H	-1.26688300	-2.45574200	-1.17895500
	H	-0.95637400	-2.54726900	1.29260500
	H	-1.54173800	-0.56631700	2.70855200
	H	-2.71630700	0.04928700	1.41716800
	H	-3.09174500	0.04614700	-0.69306400
	H	-2.19972600	-0.37819300	-2.24992900
	C	-1.02989400	2.15881800	-0.73325800
	C	0.32099600	1.42827800	1.63818400
	C	1.08494300	1.69826200	0.45686400
	C	0.39554100	2.08371900	-0.74348600
	H	0.95591000	2.14303000	-1.68452900
	H	2.15113000	1.44950500	0.40148200
	H	0.84988300	0.97732900	2.48768400
	H	-0.52245600	2.07345400	1.92190500
	H	-1.56336700	2.56074800	0.14048400
	H	-1.55042300	2.28840100	-1.68849700
	Tc	-0.39761300	0.09138300	-0.00925100

Table S23. The optimized geometry for the $(C_4H_6)_3Tc$ structure **Tc-3D** (C_1).

M06-L	C	-1.81304500	-1.78986800	0.27454200
	C	-2.03070300	1.42369600	0.87658800
	C	-2.20463800	0.60531900	-0.38575200
	C	-2.34797400	-0.78515800	-0.59598500
	H	-2.67647900	-1.08175300	-1.59685800
	H	-2.48197600	1.20392600	-1.25664500
	H	-2.89361500	2.08651300	1.04249500
	H	-1.96807800	0.76642600	1.75034000
	H	-1.95035200	-1.71239500	1.35547700
	H	-1.88478500	-2.81569600	-0.09263700
	C	0.41856500	-0.73371400	1.62649200
	C	3.01723100	-0.11124100	-0.06864100
	C	1.95914600	-1.19829400	-0.24564100
	C	1.11748400	-1.68069700	0.79172700
	H	0.81449500	-2.72670900	0.80113900
	H	2.26237600	-1.97016100	-0.95817900
	H	3.57884300	-0.04028000	-1.00972700
	H	3.75284000	-0.39733800	0.69813800
	H	0.96123400	0.13220400	2.01057200
	H	-0.29235700	-1.13422700	2.35221700
	C	-0.71503800	2.24968700	0.73793800
	C	2.41238600	1.26012500	0.21352600
	C	1.28185800	1.44263100	-0.75284600
	C	-0.01281100	1.88209900	-0.55535900
	H	-0.54331400	2.19255800	-1.45598600
	H	1.59401500	1.36604300	-1.79810300
	H	3.16099900	2.05339800	0.06659600
	H	2.07690900	1.36059900	1.25016800
	H	-0.05667300	2.06028000	1.59197900
	H	-0.91836900	3.32964000	0.74104900
	Tc	-0.21001100	-0.46652400	-0.39515300

BP86	C	-1.74748700	-1.87154000	0.29516600
	C	-2.17757000	1.37953900	0.83968700
	C	-2.25088100	0.51072900	-0.41812600
	C	-2.30943300	-0.90258200	-0.61581400
	H	-2.60335200	-1.23430000	-1.62790400
	H	-2.54918000	1.07708100	-1.31600200
	H	-3.08264200	2.01603900	0.93638600
	H	-2.12459900	0.74648100	1.74168900
	H	-1.91245200	-1.78114900	1.37895600
	H	-1.74381300	-2.91345900	-0.06241700
	C	0.47461000	-0.70019900	1.64696300
	C	3.07148800	-0.00802800	-0.10179200
	C	2.03285900	-1.13810300	-0.25091500
	C	1.20763100	-1.63883700	0.80702400
	H	0.94284100	-2.70487500	0.83304400
	H	2.33810200	-1.90954300	-0.97782300
	H	3.60115700	0.08996100	-1.07016500
	H	3.84396400	-0.27667100	0.64927300
	H	0.99037900	0.19660400	2.01857300
	H	-0.22238500	-1.12390200	2.38546100
	C	-0.87567500	2.26171500	0.72488700
	C	2.40718700	1.34770800	0.21412000
	C	1.22067900	1.44248800	-0.72273200
	C	-0.11473400	1.84435000	-0.54167300
	H	-0.62018200	2.15679200	-1.46819400
	H	1.52968900	1.38256400	-1.78122500
	H	3.11074500	2.18709900	0.02728000
	H	2.11102000	1.42589600	1.27367400
	H	-0.24841200	2.13265200	1.62275100
	H	-1.12157800	3.34174100	0.65981300
	Tc	-0.18305500	-0.46447600	-0.38334700

Table S24. The optimized geometry for the $(C_4H_6)_3Mo$ structure **Mo-1S** (C_{3h}).

M06-L	C	-1.69969800	0.57278800	1.39140500
	C	-1.69969800	0.57278800	-1.39140500
	C	-2.15215000	-0.59557300	-0.70090000
	C	-2.15215000	-0.59557300	0.70090000
	H	-2.33234200	-1.52992400	1.22822400
	H	-2.33234200	-1.52992400	-1.22822400
	H	-1.57715000	0.50216300	-2.47041900
	H	-2.09115600	1.53458700	-1.05313300
	H	-2.09115600	1.53458700	1.05313300
	H	-1.57715000	0.50216300	2.47041900
	C	0.35380000	-1.75837600	1.39140500
	C	0.35380000	-1.75837600	-1.39140500
	C	1.59185600	-1.56603000	-0.70090000
	C	1.59185600	-1.56603000	0.70090000
	H	2.49112400	-1.25490600	1.22822400
	H	2.49112400	-1.25490600	-1.22822400
	H	0.35368900	-1.61693300	-2.47041900
	H	-0.28341400	-2.57828800	-1.05313300
	H	-0.28341400	-2.57828800	1.05313300
	H	0.35368900	-1.61693300	2.47041900
	C	1.34589800	1.18558700	1.39140500
	C	1.34589800	1.18558700	-1.39140500
	C	0.56029400	2.16160300	-0.70090000
	C	0.56029400	2.16160300	0.70090000
	H	-0.15878200	2.78483000	1.22822400
	H	-0.15878200	2.78483000	-1.22822400
	H	1.22346100	1.11477100	-2.47041900
	H	2.37457000	1.04370100	-1.05313300
	H	2.37457000	1.04370100	1.05313300
	H	1.22346100	1.11477100	2.47041900
	Mo	0.00000000	0.00000000	0.00000000

BP86	C	-1.69969800	0.57278800	1.39140500
	C	-1.69969800	0.57278800	-1.39140500
	C	-2.15215000	-0.59557300	-0.70090000
	C	-2.15215000	-0.59557300	0.70090000
	H	-2.33234200	-1.52992400	1.22822400
	H	-2.33234200	-1.52992400	-1.22822400
	H	-1.57715000	0.50216300	-2.47041900
	H	-2.09115600	1.53458700	-1.05313300
	H	-2.09115600	1.53458700	1.05313300
	H	-1.57715000	0.50216300	2.47041900
	C	0.35380000	-1.75837600	1.39140500
	C	0.35380000	-1.75837600	-1.39140500
	C	1.59185600	-1.56603000	-0.70090000
	C	1.59185600	-1.56603000	0.70090000
	H	2.49112400	-1.25490600	1.22822400
	H	2.49112400	-1.25490600	-1.22822400
	H	0.35368900	-1.61693300	-2.47041900
	H	-0.28341400	-2.57828800	-1.05313300
	H	-0.28341400	-2.57828800	1.05313300
	H	0.35368900	-1.61693300	2.47041900
	C	1.34589800	1.18558700	1.39140500
	C	1.34589800	1.18558700	-1.39140500
	C	0.56029400	2.16160300	-0.70090000
	C	0.56029400	2.16160300	0.70090000
	H	-0.15878200	2.78483000	1.22822400
	H	-0.15878200	2.78483000	-1.22822400
	H	1.22346100	1.11477100	-2.47041900
	H	2.37457000	1.04370100	-1.05313300
	H	2.37457000	1.04370100	1.05313300
	H	1.22346100	1.11477100	2.47041900
	Mo	0.00000000	0.00000000	0.00000000

Table S25. The optimized geometry for the $(C_4H_6)_3Mo$ structure **Mo-2S** (C_1).

M06-L	C	0.76138500	-1.04145000	-1.40352900
	C	3.57377200	-0.87767900	-0.17682600
	C	2.47454900	-1.29442800	0.48015400
	C	1.12950600	-1.46557800	-0.07605700
	H	0.63623100	-2.36299600	0.30199200
	H	2.58148500	-1.55490800	1.53730900
	H	4.53348000	-0.78848500	0.32437600
	H	3.54595000	-0.62874400	-1.23703300
	H	1.49572400	-0.47743400	-1.97769800
	H	0.16705300	-1.70623000	-2.02781300
	C	-2.34136900	0.03756200	-1.09203900
	C	-1.76110500	-0.54724300	1.66649900
	C	-1.78724100	-1.57540000	0.68990000
	C	-2.06142400	-1.28992000	-0.66416300
	H	-1.86078700	-2.06922200	-1.39513200
	H	-1.38751300	-2.55831000	0.92960400
	H	-1.42273100	-0.79240800	2.66881300
	H	-2.51939900	0.24260500	1.63948700
	H	-2.99708700	0.67053500	-0.48497100
	H	-2.42801700	0.22124000	-2.15904800
	C	-0.26218400	2.07876900	-1.11593100
	C	0.27416500	1.54906400	1.65424400
	C	1.31907400	1.55477600	0.70008700
	C	1.05821800	1.79642300	-0.66587500
	H	1.85269700	1.57527600	-1.37533100
	H	2.29057200	1.14186100	0.95822800
	H	0.49624800	1.23723600	2.67034500
	H	-0.53392700	2.28576300	1.58451100
	H	-0.89299100	2.75838500	-0.53338400
	H	-0.43510600	2.13943800	-2.18663500
	Mo	-0.41395100	0.16945300	0.01865700

BP86	C	0.79763300	-0.97219500	-1.45503400
	C	3.67350100	-0.84284700	-0.20780700
	C	2.54793300	-1.25440600	0.43817900
	C	1.19633100	-1.42817800	-0.13512300
	H	0.72028100	-2.35214800	0.22501600
	H	2.64360000	-1.53299000	1.50201100
	H	4.63406400	-0.77434600	0.31668400
	H	3.66646000	-0.58756000	-1.27540400
	H	1.50065100	-0.34966900	-2.02255600
	H	0.21368300	-1.64367700	-2.09822100
	C	-2.38504200	0.01264100	-1.06379000
	C	-1.70310700	-0.65936200	1.68898200
	C	-1.74176700	-1.66178600	0.66640000
	C	-2.06601800	-1.33377300	-0.68123400
	H	-1.87958200	-2.09445800	-1.44959100
	H	-1.32684100	-2.65848000	0.86428600
	H	-1.31000900	-0.94191500	2.67213300
	H	-2.48811700	0.11560000	1.73213900
	H	-3.04973100	0.62198000	-0.42681400
	H	-2.49227700	0.22396300	-2.13391800
	C	-0.39773700	2.14405600	-1.03723700
	C	0.27468800	1.49382100	1.70702900
	C	1.28988700	1.58552000	0.70418700
	C	0.96115800	1.89811200	-0.64703600
	H	1.74148400	1.76218500	-1.40546800
	H	2.29476100	1.19644400	0.90241600
	H	0.55808800	1.11514000	2.69557300
	H	-0.55104500	2.22625600	1.73258500
	H	-1.04914000	2.76671100	-0.39969700
	H	-0.61150500	2.24295100	-2.10770700
	Mo	-0.42618100	0.16129500	0.01932000

Table S26. The optimized geometry for the $(C_4H_6)_3Mo$ structure **Mo-3S** (C_1).

M06-L	C	-0.15948200	1.63135200	1.61758000
	C	0.18503500	1.86137600	-1.33774600
	C	-1.06282300	1.90275300	-0.67674400
	C	-1.23041600	1.81353300	0.72724400
	H	-2.24586400	1.65371200	1.08725800
	H	-1.96545500	1.78403200	-1.27486300
	H	0.17417600	1.82869100	-2.42477600
	H	1.03399300	2.38847600	-0.90311300
	H	0.76846300	2.17841800	1.46475100
	H	-0.39246300	1.40922600	2.65613800
	C	-0.42391100	-2.21660000	0.56833700
	C	-1.60592900	-0.92778400	-1.43425900
	C	-2.19306100	-0.78512700	-0.15304300
	C	-1.54213900	-1.40305200	0.94568400
	H	-1.88350100	-1.24702700	1.96732300
	H	-3.01015900	-0.08583600	0.01768200
	H	-1.99697900	-0.31662300	-2.24680000
	H	-1.19449800	-1.88241400	-1.74916600
	H	-0.54036900	-2.87029900	-0.29876600
	H	0.15786100	-2.67054100	1.36686700
	C	1.65648600	-0.56375000	1.45535200
	C	1.65375900	-1.18097300	-1.30816200
	C	2.24381100	0.01290000	-0.85876300
	C	2.22214400	0.35960700	0.50812400
	H	2.54986500	1.35936000	0.79279700
	H	2.52970700	0.77849300	-1.57792100
	H	1.52167700	-1.32512000	-2.37715800
	H	1.76597200	-2.08998500	-0.72403100
	H	1.94944800	-1.61007900	1.35453600
	H	1.60111900	-0.23821600	2.49181600
	Mo	0.01705200	-0.04927800	0.00132800

BP86	C	-0.10640500	1.63490400	1.64948600
	C	0.23764100	1.87727900	-1.34174200
	C	-1.01411400	1.95939300	-0.66363700
	C	-1.18022300	1.86726700	0.75186300
	H	-2.20995600	1.75997000	1.11941700
	H	-1.93255800	1.89601600	-1.26286200
	H	0.20957200	1.83795700	-2.43778300
	H	1.11251400	2.38711600	-0.91864000
	H	0.84534600	2.16199800	1.51271700
	H	-0.35826500	1.40184600	2.69153400
	C	-0.47964700	-2.23495400	0.55398900
	C	-1.64747700	-0.88462700	-1.44721000
	C	-2.23170700	-0.74020300	-0.15014900
	C	-1.58739100	-1.39396700	0.94623600
	H	-1.92950100	-1.25037100	1.97934400
	H	-3.05402800	-0.03606800	0.03158300
	H	-2.02875100	-0.25039400	-2.25831700
	H	-1.25121800	-1.85063000	-1.77597600
	H	-0.60991900	-2.87954400	-0.32861700
	H	0.09312600	-2.71522000	1.35569100
	C	1.64841400	-0.64581200	1.46520600
	C	1.60227200	-1.18709200	-1.36594900
	C	2.26105300	-0.03234700	-0.86231000
	C	2.26284800	0.27305400	0.52552600
	H	2.65114700	1.25273700	0.83790300
	H	2.60185800	0.74391800	-1.56033200
	H	1.45487400	-1.26197900	-2.44984800
	H	1.68444000	-2.14138500	-0.83635700
	H	1.90281600	-1.70940900	1.35727200
	H	1.59654400	-0.32243800	2.51235100
	Mo	0.01500900	-0.04717800	0.00150200

Table S27. The optimized geometry for the $(C_4H_6)_3Mo$ structure **Mo-1T** (C_1).

M06-L	C	2.47879200	0.98572900	0.08575800
	C	-0.09344600	1.62586700	-1.57120200
	C	0.18694500	2.13781300	-0.27270900
	C	1.18009300	1.59827600	0.56071300
	H	1.16485900	1.91008200	1.60705500
	H	-0.57280200	2.76153300	0.20061500
	H	-0.95091100	2.03549000	-2.10129500
	H	0.72723000	1.32395200	-2.22060200
	H	2.45936700	0.86780800	-1.00442200
	H	3.31668000	1.66927500	0.29471500
	C	2.71990300	-0.35603100	0.77648500
	C	0.90829400	-1.37204600	-1.63493100
	C	0.99758100	-1.96571600	-0.35457100
	C	1.51099500	-1.28856200	0.77867000
	H	1.36179600	-1.81523600	1.72062900
	H	0.37300300	-2.84128400	-0.16155200
	H	0.38302300	-1.90795400	-2.42101700
	H	1.70730000	-0.71820900	-1.98701800
	H	3.59640100	-0.86079400	0.34253100
	H	2.97477600	-0.15561000	1.82446200
	C	-2.33039900	-0.98115700	-0.71464800
	C	-1.32655700	-0.43928400	1.90420500
	C	-2.09177800	0.54667300	1.21026900
	C	-2.57767500	0.27923400	-0.09019200
	H	-2.96982900	1.11165500	-0.67434500
	H	-2.14820100	1.56795800	1.58584800
	H	-0.86127500	-0.14896000	2.84409900
	H	-1.65418900	-1.48155900	1.87767500
	H	-2.43574400	-1.89242000	-0.12017500
	H	-2.61857200	-1.09306200	-1.75749100
	Mo	-0.31498600	-0.11803500	-0.09325700

BP86	C	2.52664700	0.98134100	0.07560300
	C	-0.10399200	1.64051800	-1.58401300
	C	0.19939900	2.14767900	-0.27553600
	C	1.20927600	1.58990700	0.55298900
	H	1.20221100	1.90697600	1.60779600
	H	-0.54206900	2.80195600	0.20646300
	H	-0.98270500	2.05279800	-2.09648100
	H	0.70301800	1.32764100	-2.25829700
	H	2.52609200	0.88055000	-1.02472600
	H	3.36751600	1.66757100	0.31944700
	C	2.75685300	-0.38706000	0.75557700
	C	0.84971200	-1.39223200	-1.65700800
	C	0.96174900	-1.98428700	-0.36251500
	C	1.51576700	-1.30374600	0.76685900
	H	1.37109700	-1.83170700	1.71973800
	H	0.33980500	-2.86992200	-0.15388800
	H	0.28576900	-1.92803200	-2.42969300
	H	1.64937400	-0.74354400	-2.03903500
	H	3.62235000	-0.90900800	0.29453800
	H	3.03018800	-0.20314300	1.81248100
	C	-2.34722000	-0.96960700	-0.73551400
	C	-1.31214400	-0.44337000	1.93592700
	C	-2.10444300	0.53856700	1.24332800
	C	-2.60073900	0.27934800	-0.07104000
	H	-3.01957800	1.12256600	-0.63849200
	H	-2.18587200	1.56196000	1.63590000
	H	-0.83762100	-0.13973100	2.87790600
	H	-1.61204200	-1.50292800	1.90928600
	H	-2.41675000	-1.91289700	-0.17142800
	H	-2.62940800	-1.05031800	-1.79260200
	Mo	-0.31372800	-0.10507500	-0.08683000

Table S28. The optimized geometry for the $(C_4H_6)_3Mo$ structure **Mo-2T** (C_1).

M06-L	C	1.94097800	-0.63023600	1.45241700
	C	1.15405200	-1.90125100	-0.94518200
	C	2.12887900	-0.86284200	-0.98608800
	C	2.53565900	-0.23495300	0.21758500
	H	3.15548600	0.65825300	0.15874900
	H	2.43630000	-0.41985100	-1.93308600
	H	0.78495800	-2.27343200	-1.89914700
	H	1.22113700	-2.66699100	-0.16784900
	H	1.78330000	-1.69085500	1.65433700
	H	2.17240000	-0.03849600	2.33603100
	C	-0.86509100	1.64389900	1.11322300
	C	-0.11642300	1.46954500	-1.62945100
	C	0.75449100	2.08303200	-0.68545000
	C	0.39099500	2.14840900	0.68434000
	H	1.15815700	2.42633600	1.40688400
	H	1.77757600	2.33144500	-0.96351300
	H	0.25229100	1.32501100	-2.64294400
	H	-1.19332600	1.63622600	-1.55539100
	H	-1.75030500	1.82778500	0.50085200
	H	-1.05641500	1.61053800	2.18379000
	C	-1.22687900	-1.38394000	1.24081100
	C	-3.59826200	0.14130900	-0.05812100
	C	-2.71877300	-0.67308100	-0.69439000
	C	-1.68994800	-1.48428700	-0.07972900
	H	-1.32871300	-2.31349000	-0.68175800
	H	-2.80225500	-0.76870800	-1.77823300
	H	-4.34978000	0.69866000	-0.60929600
	H	-3.59302400	0.25293600	1.02457600
	H	-1.76256400	-0.78436300	1.97185900
	H	-0.64452000	-2.20918800	1.64752200
	Mo	0.27622000	-0.03560600	0.03730500

BP86	C	1.97647700	-0.63760100	1.45299100
	C	1.10197300	-1.93960500	-0.95843500
	C	2.11137200	-0.92086500	-1.01349400
	C	2.55892700	-0.28292300	0.18639000
	H	3.21551000	0.59307700	0.09934800
	H	2.42619300	-0.49795600	-1.97779500
	H	0.70051900	-2.29663900	-1.91611800
	H	1.14428100	-2.71013900	-0.17265100
	H	1.78726200	-1.69371500	1.69346100
	H	2.23517300	-0.01838700	2.32205200
	C	-0.81906900	1.70532600	1.13230100
	C	-0.12920700	1.43864300	-1.67266900
	C	0.76462600	2.07862300	-0.75119300
	C	0.43104200	2.19070000	0.63748300
	H	1.22129900	2.50322400	1.33447400
	H	1.78825000	2.32864500	-1.06115400
	H	0.23345100	1.25054000	-2.69123900
	H	-1.21402600	1.59568600	-1.59061600
	H	-1.73709200	1.84022200	0.54297400
	H	-0.96533600	1.69919300	2.21948200
	C	-1.23276900	-1.26459100	1.33578500
	C	-3.66277700	0.12053700	-0.11851200
	C	-2.73066900	-0.71099900	-0.68354000
	C	-1.68028100	-1.46414400	0.00144400
	H	-1.34478200	-2.36544500	-0.52331000
	H	-2.79069700	-0.88201300	-1.77003900
	H	-4.42466500	0.61257400	-0.73408900
	H	-3.70046700	0.29899900	0.96302600
	H	-1.79138000	-0.62072600	2.02216000
	H	-0.66562600	-2.07368600	1.81583200
	Mo	0.27962500	-0.03433400	0.05078300

Table S29. The optimized geometry for the $(C_4H_6)_3Mo$ structure **Mo-3T** (C_1).

M06-L	C	-1.91039500	-1.84028500	0.22246400
	C	-2.08926700	1.32529700	0.89490900
	C	-2.33292100	0.57191200	-0.40365700
	C	-2.46048900	-0.81359200	-0.59809600
	H	-2.75257800	-1.10143000	-1.61137600
	H	-2.65395400	1.19101100	-1.24052600
	H	-2.95765800	1.95404900	1.13733900
	H	-1.99528800	0.61678700	1.72766400
	H	-1.94895100	-1.73456800	1.30879600
	H	-2.02832600	-2.86400800	-0.12638800
	C	0.67899200	-1.04642700	1.57286300
	C	3.04947900	-0.00491900	-0.06127100
	C	2.02938800	-1.08508000	-0.44440400
	C	1.27043400	-1.81625500	0.50901800
	H	0.98582100	-2.85169500	0.33166200
	H	2.29738600	-1.65077000	-1.34038600
	H	3.71300600	0.14329900	-0.92273200
	H	3.69641300	-0.34061000	0.76231700
	H	1.28853700	-0.27602800	2.05390800
	H	0.01513400	-1.56277400	2.26732900
	C	-0.79015600	2.18486800	0.78320200
	C	2.36810600	1.32439800	0.25441200
	C	1.24327300	1.47190200	-0.73224100
	C	-0.08320000	1.86275400	-0.52091900
	H	-0.61494800	2.20369300	-1.41128600
	H	1.57362500	1.48145700	-1.77373200
	H	3.07367200	2.16699300	0.17880700
	H	1.98811300	1.34716600	1.28255900
	H	-0.13109400	1.97509400	1.63387600
	H	-1.01965000	3.25872700	0.83426200
	Mo	-0.19925500	-0.39913900	-0.33213700

BP86	C	-1.88129200	-1.89630800	0.22194700
	C	-2.17053100	1.32880500	0.87114000
	C	-2.34789400	0.52854000	-0.43018500
	C	-2.44267400	-0.87761200	-0.61914400
	H	-2.71864100	-1.18105000	-1.64345800
	H	-2.67746400	1.12913800	-1.29004700
	H	-3.06292000	1.96616200	1.04209100
	H	-2.10813100	0.64739600	1.73883500
	H	-1.93242400	-1.79706500	1.31620000
	H	-1.94875800	-2.93023600	-0.14120800
	C	0.69031700	-1.00315500	1.60244800
	C	3.09908200	0.01568200	-0.09908700
	C	2.06736600	-1.09121900	-0.43043000
	C	1.30298500	-1.79797000	0.54964000
	H	1.02769400	-2.85060000	0.40128300
	H	2.32090400	-1.68375800	-1.32510500
	H	3.72207600	0.16668300	-1.00204100
	H	3.79105800	-0.31097700	0.70455700
	H	1.28769700	-0.20205600	2.06589300
	H	0.03415100	-1.52273500	2.31566700
	C	-0.86052800	2.20960500	0.78330600
	C	2.40808200	1.35381600	0.24207700
	C	1.23571200	1.46811700	-0.71783000
	C	-0.11289800	1.85701200	-0.51066600
	H	-0.62583500	2.21501800	-1.41778900
	H	1.55513300	1.48970300	-1.77371100
	H	3.10652100	2.21030300	0.12029400
	H	2.07270100	1.37787300	1.29433200
	H	-0.22501100	2.02530200	1.66663700
	H	-1.10883000	3.29120100	0.79230700
	Mo	-0.20086400	-0.39552800	-0.32485800

Table S30. The optimized geometry for the $(C_4H_6)_3Nb$ structure **Nb-1D** (C_1).

M06-L	C	-0.01776200	1.74518200	1.60276700
	C	0.29547900	1.98780700	-1.24393100
	C	-0.98272500	2.02869100	-0.63944300
	C	-1.14328900	1.91902500	0.76304500
	H	-2.15099200	1.78612400	1.15530800
	H	-1.87330400	1.95337700	-1.26257600
	H	0.34891200	1.97124200	-2.33010200
	H	1.13523100	2.47527800	-0.74826000
	H	0.89401000	2.30281900	1.38857400
	H	-0.18997000	1.54850100	2.65896700
	C	-0.70480400	-2.20828800	0.84684600
	C	-1.53505200	-0.98070200	-1.54748100
	C	-2.24344100	-0.72834100	-0.35380900
	C	-1.80530100	-1.32326800	0.86273500
	H	-2.25168600	-1.00251400	1.80315400
	H	-3.02003800	0.03324400	-0.31487600
	H	-1.80185700	-0.42265800	-2.44247300
	H	-1.11109400	-1.96486400	-1.73303000
	H	-0.53088300	-2.84721300	-0.01881500
	H	-0.33482900	-2.59860600	1.78927600
	C	1.76703600	-0.82565200	1.39755900
	C	1.57463400	-1.24664700	-1.40304500
	C	2.26020000	-0.13155000	-0.89801400
	C	2.33113300	0.11909900	0.49603000
	H	2.71017800	1.08068300	0.83752800
	H	2.59075600	0.65007800	-1.57853900
	H	1.43942600	-1.34309900	-2.47610400
	H	1.55703400	-2.17788900	-0.84014600
	H	1.90772400	-1.88556800	1.18873200
	H	1.73749800	-0.57676800	2.45583000
	Nb	0.00407900	-0.02717800	0.02848800

BP86	C	-0.06580400	1.71249100	1.66487400
	C	0.38799700	2.03415500	-1.22458500
	C	-0.91576000	2.08039800	-0.64840500
	C	-1.13998700	1.94161200	0.75638500
	H	-2.17906500	1.83640800	1.09836800
	H	-1.79343600	2.06222800	-1.30917000
	H	0.46374600	2.03195300	-2.31907900
	H	1.23452800	2.49541200	-0.69924200
	H	0.88651700	2.24201000	1.53086400
	H	-0.31568200	1.47421100	2.70674000
	C	-0.79703800	-2.16615000	0.93388500
	C	-1.48108800	-1.03597900	-1.61390800
	C	-2.24531300	-0.71041200	-0.45853000
	C	-1.88286500	-1.25270700	0.82025900
	H	-2.39025700	-0.87921700	1.72058200
	H	-3.02810600	0.05815900	-0.49958100
	H	-1.69299700	-0.50291100	-2.54953300
	H	-1.06771600	-2.04295800	-1.74345500
	H	-0.56100600	-2.86182800	0.11753000
	H	-0.49616700	-2.49865800	1.93269100
	C	1.70744600	-0.90031300	1.43718100
	C	1.59168100	-1.25205700	-1.41269800
	C	2.30123700	-0.16253100	-0.84782500
	C	2.33110300	0.05499200	0.56431900
	H	2.74742800	0.99707700	0.94501800
	H	2.69689200	0.62412900	-1.50274600
	H	1.48607700	-1.30562200	-2.50173200
	H	1.53481600	-2.21182800	-0.88404200
	H	1.84143700	-1.96891900	1.22241400
	H	1.64443200	-0.65991400	2.50527000
	Nb	0.00582700	-0.02318900	0.00983900

Table S31. The optimized geometry for the $(C_4H_6)_3Nb$ structure **Nb-2D** (C_1).

M06-L	C	-0.93848800	-1.26917400	1.58565300
	C	-2.99019200	0.74059900	0.12693500
	C	-2.48556600	-0.62545900	-0.31023100
	C	-1.83904500	-1.56584900	0.49984600
	H	-1.79554800	-2.58489500	0.09918500
	H	-2.95611100	-1.03810800	-1.20280300
	H	-3.68809100	1.10232600	-0.63697200
	H	-3.56896400	0.66597300	1.05853600
	H	-1.16013900	-0.41060400	2.22708900
	H	-0.53916600	-2.11967900	2.13705900
	C	1.99461000	-1.32001000	-1.22960000
	C	2.56015000	1.24013800	0.64812100
	C	2.10004500	-0.15576300	0.99994400
	C	2.18813000	-1.29281100	0.19405200
	H	3.14081000	1.21270600	-0.28585400
	H	3.23661600	1.61387100	1.42876400
	C	-1.83603300	1.73187100	0.26278300
	C	1.36106800	2.16766000	0.44797400
	C	0.56548200	1.57682600	-0.68822800
	C	-0.85389200	1.46420600	-0.85967900
	H	-1.26638400	1.57367400	-1.86551300
	H	1.12464000	1.57595200	-1.63496100
	H	1.67966600	3.20254800	0.24134000
	H	0.76100100	2.20199900	1.36874200
	H	-1.33886500	1.58683000	1.23443700
	H	-2.18484500	2.77710800	0.27284300
	H	1.97443300	-0.34854900	2.06648700
	H	2.37603900	-0.48134800	-1.81997600
	H	2.09606200	-2.28211000	-1.72815000
	H	2.04678400	-2.24888800	0.70829900
	Nb	0.02693800	-0.54029800	-0.33497500

BP86	C	-0.89208200	-1.28904800	1.60186900
	C	-3.03803800	0.70524200	0.13306000
	C	-2.47844500	-0.65838800	-0.30070100
	C	-1.80461100	-1.60044900	0.51272200
	H	-1.76194100	-2.63288400	0.11870100
	H	-2.93760600	-1.08648100	-1.20414900
	H	-3.75655400	1.03406700	-0.64119500
	H	-3.61255200	0.61754500	1.07790000
	H	-1.10971800	-0.42709700	2.25406300
	H	-0.46272000	-2.14309000	2.14413700
	C	1.99844600	-1.34299800	-1.23963800
	C	2.57030600	1.28427700	0.64250300
	C	2.09827000	-0.12630800	0.99161400
	C	2.20781400	-1.28945300	0.19246200
	H	3.16439700	1.26207600	-0.29321700
	H	3.24092600	1.66036600	1.44218500
	C	-1.90671600	1.74942200	0.25410300
	C	1.35657600	2.22007500	0.43861800
	C	0.53769300	1.59840300	-0.68502400
	C	-0.89263400	1.46808400	-0.85639400
	H	-1.29763200	1.55719400	-1.87776800
	H	1.09279300	1.59516200	-1.64610100
	H	1.68104800	3.25551100	0.19172100
	H	0.76985400	2.28027500	1.37575300
	H	-1.42059400	1.65796200	1.24590800
	H	-2.29490300	2.79058900	0.20612300
	H	1.98147100	-0.31684000	2.06898700
	H	2.36489000	-0.51602500	-1.87004400
	H	2.06824300	-2.32765700	-1.72189100
	H	2.10278900	-2.25057700	0.72519400
	Nb	0.04020300	-0.54447000	-0.33432900

Table S32. The optimized geometry for the $(C_4H_6)_3Zr$ structure **Zr-1S** (C_1).

M06-L	C	0.25061700	-1.71735900	-1.56108500
	C	2.61409400	-1.07793700	0.30157300
	C	1.28765900	-1.71694300	0.69591500
	C	0.38847800	-2.22961800	-0.23910300
	H	-0.42047600	-2.85215200	0.14380600
	H	1.20864200	-2.09062700	1.71530800
	H	3.26874000	-1.04997900	1.17947600
	H	3.12375400	-1.70097200	-0.44825000
	H	1.15864600	-1.43299300	-2.10065800
	H	-0.51383300	-2.15783600	-2.19766200
	C	-2.45247200	0.66095600	-0.88425200
	C	-1.48784700	0.25522300	1.83839500
	C	-2.13216700	-0.79876100	1.09230200
	C	-2.58863400	-0.60352000	-0.21554300
	H	-2.86056100	-1.48720700	-0.79345500
	H	-2.09945000	-1.81811000	1.47582300
	H	-1.09917200	-0.00214700	2.82129800
	H	-1.94506500	1.25141200	1.80893800
	H	-2.69428500	1.56612500	-0.32057600
	H	-2.75966100	0.71392600	-1.92600900
	C	2.46396000	0.34818300	-0.23196900
	C	0.32179000	2.39072400	-0.93312700
	C	0.92794700	2.29173900	0.34207800
	C	1.74832000	1.24022500	0.75057800
	H	2.06057200	1.17536200	1.79003700
	H	0.50148800	2.90033000	1.14241000
	H	-0.39289800	3.18679700	-1.11430500
	H	0.87979700	2.08829300	-1.82317000
	H	1.91322500	0.32163700	-1.19698200
	H	3.43127600	0.77146400	-0.54411000
	Zr	-0.27028000	0.15898000	-0.13366200

BP86	C	0.23102400	-1.60970000	-1.64012400
	C	2.67947300	-1.11196500	0.31603400
	C	1.30951900	-1.74088500	0.63010700
	C	0.39929800	-2.20405100	-0.34468200
	H	-0.40046300	-2.87205200	0.00635700
	H	1.20921700	-2.18516000	1.62958700
	H	3.27556000	-1.11435300	1.24803300
	H	3.23062600	-1.74071600	-0.41474700
	H	1.11792000	-1.23606200	-2.17782700
	H	-0.54886400	-2.01404000	-2.29670800
	C	-2.46295700	0.69343200	-0.90876000
	C	-1.55590500	0.19911000	1.87148200
	C	-2.21146200	-0.82452000	1.06942400
	C	-2.63674100	-0.58562000	-0.25786900
	H	-2.92166700	-1.45803500	-0.86349500
	H	-2.21882800	-1.86208700	1.43108200
	H	-1.17625200	-0.10935900	2.85412300
	H	-1.98923600	1.21603800	1.87895500
	H	-2.68878900	1.61166000	-0.34336500
	H	-2.72281400	0.76476200	-1.97216100
	C	2.60438600	0.34328800	-0.20146900
	C	0.31742100	2.36130500	-0.95753500
	C	0.95976900	2.27920400	0.31408800
	C	1.79158900	1.22413200	0.74464800
	H	2.09880000	1.20160100	1.79841900
	H	0.54646400	2.91587900	1.11341700
	H	-0.39320500	3.17286500	-1.14330300
	H	0.82339500	1.99139900	-1.86480900
	H	2.17347600	0.35600700	-1.22677800
	H	3.62420400	0.76398900	-0.34378400
	Zr	-0.28980100	0.16138200	-0.07812600

Table S33. The optimized geometry for the $(C_4H_6)_3Zr$ structure **Zr-2S** (C_1).

M06-L	C	-0.62988600	1.79189400	1.47562000
	C	0.62054800	2.10888400	-1.04022500
	C	-0.80200200	2.03920700	-0.95616200
	C	-1.42015600	1.92154000	0.31352700
	H	-2.49718000	1.76650000	0.35428400
	H	-1.41497600	1.95494700	-1.85373200
	H	1.06385800	2.11102700	-2.03530900
	H	1.17444200	2.69510800	-0.30519100
	H	0.31022000	2.33704800	1.55556200
	H	-1.12147800	1.57781800	2.42224400
	C	-1.09826900	-1.68997000	1.39596800
	C	-1.09022400	-1.59223700	-1.57393100
	C	-2.11952200	-1.03308600	-0.78247100
	C	-2.11906000	-1.08908100	0.63578000
	H	-2.85616900	-0.48075900	1.15762500
	H	-2.85515500	-0.38005200	-1.25064100
	H	-1.13205100	-1.47669700	-2.65303900
	H	-0.59990200	-2.50896600	-1.24318100
	H	-0.53276500	-2.52584700	0.97752100
	H	-1.14170800	-1.66603600	2.47910700
	C	1.64842700	-0.73910600	1.68286600
	C	1.95281100	-1.32840500	-1.13295700
	C	2.48513300	-0.17301500	-0.56267300
	C	2.31244000	0.14774200	0.81840800
	H	2.58939400	1.14499900	1.15430700
	H	2.93420500	0.58138500	-1.20415200
	H	2.06148600	-1.52368900	-2.19442300
	H	1.73807800	-2.20147900	-0.51481200
	H	1.72633500	-1.81361400	1.51941500
	H	1.46148800	-0.44353900	2.71123200
	Zr	0.01626100	-0.03335800	-0.06798300

BP86	C	-0.14160900	1.97597100	1.48709800
	C	0.66397900	2.01200100	-1.27125300
	C	-0.71483600	2.08929100	-0.92191500
	C	-1.10748300	2.09762300	0.45846300
	H	-2.17866400	2.07196700	0.69630900
	H	-1.49357900	2.03687100	-1.69507100
	H	0.92915000	1.89186900	-2.32956400
	H	1.42244600	2.50755600	-0.65227200
	H	0.86534900	2.39042800	1.36578700
	H	-0.47959300	1.84408400	2.52205900
	C	-1.35329700	-1.51255000	1.46838000
	C	-1.25396900	-1.55078700	-1.55047700
	C	-2.22858000	-0.83991200	-0.79897300
	C	-2.27521500	-0.82710600	0.63418600
	H	-2.97186400	-0.11980400	1.10323000
	H	-2.88711500	-0.13169900	-1.32089600
	H	-1.24379400	-1.45249600	-2.64149700
	H	-0.84719300	-2.50056700	-1.17586500
	H	-0.88454600	-2.44714300	1.12887300
	H	-1.42603000	-1.40517900	2.55527900
	C	1.56965100	-1.03274000	1.62259600
	C	1.76917200	-1.36210500	-1.25298500
	C	2.44473900	-0.30093400	-0.58544300
	C	2.33326700	-0.12074300	0.82960400
	H	2.75147900	0.79114900	1.27581700
	H	2.96615300	0.47115800	-1.16488600
	H	1.82526100	-1.42622200	-2.34550500
	H	1.63809700	-2.32843000	-0.74374200
	H	1.57753300	-2.10395900	1.37176200
	H	1.44295200	-0.81871700	2.68992500
	Zr	0.01897600	-0.07597300	-0.03388600
